# Reporting quality of interventions using a wearable activity tracker to improve physical activity in patients with inflammatory arthritis or osteoarthritis: a systematic review

**DOI:** 10.1007/s00296-022-05241-x

**Published:** 2022-12-01

**Authors:** M. A. T. van Wissen, M. A. M. Berger, J. W. Schoones, M. G. J. Gademan, C. H. M. van den Ende, T. P. M. Vliet Vlieland, S. F. E. van Weely

**Affiliations:** 1 Department of Orthopaedics, Rehabilitation and Physical Therapy, Leiden University Medical Center, Albinusdreef 2, P.O. Box 9600, 2300 RC Leiden, The Netherlands; 2 The Hague University of Applied Sciences, The Hague, The Netherlands; 3 Directorate of Research Policy (Walaeus Library), Leiden, The Netherlands; 4 Department of Clinical Epidemiology, Leiden University Medical Center, Leiden, The Netherlands; 5 Department of Research, Sint Maartenskliniek, Nijmegen, The Netherlands; 6 Department of Rheumatology, Radboud University Medical Center, Nijmegen, The Netherlands

**Keywords:** Rheumatoid arthritis, Axial spondyloarthritis, Osteoarthritis, Wearable activity tracker, Physical activity, Reporting quality

## Abstract

To assess the reporting quality of interventions aiming at promoting physical activity (PA) using a wearable activity tracker (WAT) in patients with inflammatory arthritis (IA) or hip/knee osteoarthritis (OA). A systematic search was performed in eight databases (including PubMed, Embase and Cochrane Library) for studies published between 2000 and 2022. Two reviewers independently selected studies and extracted data on study characteristics and the reporting of the PA intervention using a WAT using the Consensus on Exercise Reporting Template (CERT) (12 items) and Consolidated Standards of Reporting Trials (CONSORT) E-Health checklist (16 items). The reporting quality of each study was expressed as a percentage of reported items of the total CERT and CONSORT E-Health (50% or less = poor; 51–79% = moderate; and 80–100% = good reporting quality). Sixteen studies were included; three involved patients with IA and 13 with OA. Reporting quality was poor in 6/16 studies and moderate in 10/16 studies, according to the CERT and poor in 8/16 and moderate in 8/16 studies following the CONSORT E-Health checklist. Poorly reported checklist items included: the description of decision rule(s) for determining progression and the starting level, the number of adverse events and how adherence or fidelity was assessed. In clinical trials on PA interventions using a WAT in patients with IA or OA, the reporting quality of delivery process is moderate to poor. The poor reporting quality of the progression and tailoring of the PA programs makes replication difficult. Improvements in reporting quality are necessary.

**Supplementary Information:**

The online version contains supplementary material available at 10.1007/s00296-022-05241-x.

## Introduction

People with rheumatic and musculoskeletal diseases (RMDs) are at greater risk of physical inactivity compared with their healthy peers, as they are often limited by disabling health problems and encounter disease specific barriers to be physically active [1, 2]. Promotion of physical activity (PA) and exercise are key components in clinical practice guidelines for the management of people with RMDs [3–5], based on the favorable effects of PA on pain, disease activity, joint range of motion, aerobic capacity, muscle strength and overall functional ability [6–11]. In addition, patients with an RMD may gain from the general health benefits and from the reduction of the increased risk of cardiovascular disease associated with inflammatory RMDs [12].

A frequently used strategy to promote PA in adults with chronic diseases includes monitoring and feedback of PA [13], which can be supported by the use of wearable activity trackers (WATs). WATs to stimulate PA can range from pedometers to advanced WATs that can provide real-time feedback (i.e. Fitbit® or Garmin® watches). Several systematic literature reviews have shown that PA promotion with the use of WATs has a moderate, positive effect on PA levels in patients with various (chronic) diseases, including RMDs, compared to the control intervention without a WAT or with usual care [14–20]. A general conclusion from these reviews concerned the heterogeneity of the description of the interventions, whereas in one review the overall insufficient quality of reporting on the interventions was specifically addressed [16]. Using a WAT for PA promotion usually includes additional interventions, such as instruction on how to use the WAT and any digital applications or patient education, including behavioral change techniques such as individual goal setting. The variability and lack of information on these topics hamper the replication of studies for future research and the interpretation of the effects for, e.g. clinical guidelines. The reporting quality on WAT delivery in studies of PA promotion in patients with RMDs has not yet been systematically evaluated.

The reporting quality of trials with PA interventions using a WAT should preferably meet the requirements as defined in checklists for the reporting of PA interventions and of eHealth interventions [21–24]. Regarding PA or exercise interventions, there are various checklists available, including the Standard Protocol Items Recommendations for Interventional Trials (SPIRIT statement) [21], the Template for Intervention Description and Replication (TIDieR checklist) [23] and the Consensus on Exercise Reporting Template (CERT checklist) [24]. The latter has been widely applied to assess the quality of PA intervention descriptions in low back pain, hip osteoarthritis, fibromyalgia and juvenile idiopathic arthritis populations [25–28]. The CONSORT E-Health checklist is generally mentioned as a reporting tool for web-based and mobile health interventions [22].

To date, no study has systematically assessed the reporting quality of intervention strategies that used a WAT as part of PA promotion in RMDs. Therefore, the aim of this study is to provide an overview of the reporting quality of interventions promoting PA using a WAT in patients with inflammatory arthritis (IA) or osteoarthritis (OA), using the CERT and CONSORT E-Health reporting checklists.

## Methods

A systematic search was performed following the Preferred Reporting Items for Systematic Reviews and Meta-Analysis (PRISMA) statement [29]. This review was performed based on a prespecified study protocol that was registered in the international Prospective Register of Systematic Reviews (PROSPERO; CRD42021213408).

### Search strategy

The search was confined to studies published from 1st of January 2000 onwards, as the use of WATs was not common before that time. The final search was performed on June 27th 2022. The search strategy was developed by a trained librarian (JWS) and included MeSH terms and free text (see Online Resource 1 for complete PubMed search strategy). The following databases were used: PubMed, Embase (OVID), Web of Science, Cochrane Library, Emcare (OVID), PsycINFO (EbscoHOST), Academic Search Premier (EbscoHOST) and PEDro. Records were identified, imported to a reference list in EndNote™ version 20 [30] and subsequently into a reference system Rayyan (http://rayyan.qcri.org) [31] after removal of duplicates. No additional search for ongoing studies or unpublished data was done. The titles and abstracts of systematic reviews obtained from the search were also screened for potentially eligible studies.

### Eligibility criteria, participants and type of intervention

The selection of the studies was based on following criteria: Inclusion criteria: studies (i) published between 1^st^ of January 2000 and June 27th 2022; (ii) written in English or Dutch; (iii) including patients older than 18 years; (iv) with inflammatory arthritis (i.e. axial spondyloarthritis (axSpA), rheumatoid arthritis (RA), psoriatic arthritis (PsA), or juvenile arthritis (JIA)) or hip or knee OA (including those scheduled for or underwent total hip or total knee arthroplasty (THA or TKA)) and mixed populations of patients with RMDs; (v) describing interventions aiming to increase PA and including the use of a WAT for that purpose; a WAT was defined as an electronic device designed to be worn on the user’s body; including accelerometers, altimeters, or other sensors to track the wearer’s movements and/or biometric data; (vi) with one of the following designs: observational studies (including pilot studies, pre-post studies or case series (at least ten subjects)), experimental studies including randomized controlled trials (RCTs), quasi randomized controlled trials, controlled clinical trials, cluster randomized controlled trials and cross-over studies; (vii) availability of the full-text of the paper.

Exclusion criteria: studies (i) describing interventions using a way of self-monitoring of PA other than a WAT; (ii) a conference abstract, research letter or commentarial note or any other type of publication not being report of a clinical study.

### Study selection

Study selection was performed by two reviewers (MVW and MB) independently in two steps: first, titles and abstracts were screened and full-text papers were retrieved for studies potentially meeting the inclusion and exclusion criteria. Second, the full-text papers were assessed using the same eligibility criteria. Disagreements were resolved through discussion between the two reviewers and if agreement was not reached, a third and fourth reviewer were consulted (SVW and TVV). The titles and abstracts of systematic reviews obtained from the search were also screened. All articles included in the selected systematic reviews were checked against the same eligibility criteria, first for abstract and title, then full-text. This process was documented in a Microsoft Excel [32] screening data file, in which an overview of the eligibility criteria was provided for each screened record.

### Data extraction

Data extraction of the included studies was done by one reviewer (MVW) and verified by the second reviewer (MB). Disagreements were resolved by discussion between the two reviewers and if agreement was not reached a third and fourth reviewer were consulted (SVW and TVV). The following data were extracted on a pre-designed data extraction form:

#### General characteristics

First author, country, year of publication, study design, in- and exclusion criteria, number and characteristics of subjects (mean age (years), gender (female/male) and diagnosis).

#### Reporting quality of the WAT and related interventions

To assess the quality of reporting on the delivery of the WAT and concurrent strategies, the CERT and CONSORT E-Health checklist were applied. Some items of the CERT and CONSORT E-Health checklist overlap. Similarities between CERT and CONSORT E-Health items are shown in Online Resource 2.

The CERT checklist is an extension of the TIDieR checklist [23], with the aim of providing authors direction for reporting exercise interventions by including key items that are considered essential for replicating. The CERT checklist comprises 16 items listed under seven categories: what (materials); who (provider); how (delivery); where (location); when and how much (dosage); tailoring (what, how); and how well (compliance/planned and actual). The CONSORT E-Health checklist is a detailed sub-checklist as an extension to the CONSORT item 5 intervention statement [33]. It comprises 12 items, listing required and desired reporting elements characterizing the functional components and other important features of the E-Health interventions.

Data were extracted for all CERT en CONSORT E-Health items and scored ‘1’ (adequately reported) or ‘0’ (not adequately reported or unclear).

### Risk of bias assessment

Methodological quality assessment of RCTs was based on a risk of bias (RoB) assessment performed by two independent reviewers (XXX and XX). Any discrepancies were resolved by discussion and if agreement was not reached a third and fourth reviewer were consulted (XXX or XXX). The RoB 2 tool developed by the Cochrane Collaboration [34] was used for RCTs and ROBINS-I (the Risk Of Bias In Non-Randomized Studies of Interventions) was used for the non-randomized observational studies [35].

The RoB 2 tool comprises five domains, focusing on randomization process, deviations from intended interventions, missing outcome data, measurement of the outcome and selection of the reported results. The ROBINS-I includes seven domains, focusing on confounding, selection of participants into the study, classification on interventions, deviations from intended interventions, missing data, measurements of outcomes and selection of the reported results.

For both the RoB 2 and ROBINS-I tool, the judgement of the risk of bias are calculated by the individual scores of the domains, based on answers to the signaling questions. The response options of the signaling questions are: “Yes”; “Probably yes”; “Probably no”; “No”; and “No information”. Some signaling questions are only answered if the response to a previous signaling question is “Yes” or “Probably yes” (or “No” or “Probably no”). Judgment of the overall risk of bias can be low, some concern or high, with a low risk corresponding to a high-quality trial.

### Statistical analyses and synthesis

A descriptive analysis was used to assess the reporting quality. For each study, each item of the CERT and CONSORT E-Health is scored with ‘1’ (adequately reported) or ‘0’ (not adequately reported or unclear). The reporting quality of each individual study is based on the percentage of adequately reported items of the 16 items for the CERT and 12 items for the CONSORT E-Health, respectively. Based on this percentage, the overall quality of reporting is classified into poor (50% or less), moderate (51 to 79%) or good (80–100%), as described by Mercieca-Bebber et al. [36]. The reporting quality of the individual items for the CERT and CONSORT E-Health is also calculated. The reporting quality of each item was calculated by dividing the number of studies that adequately reported the item by the total number of studies included. Thus, these results are expressed as the percentage of studies reporting a specific item.

## Results

### Selection of studies

**Fig. 1:**
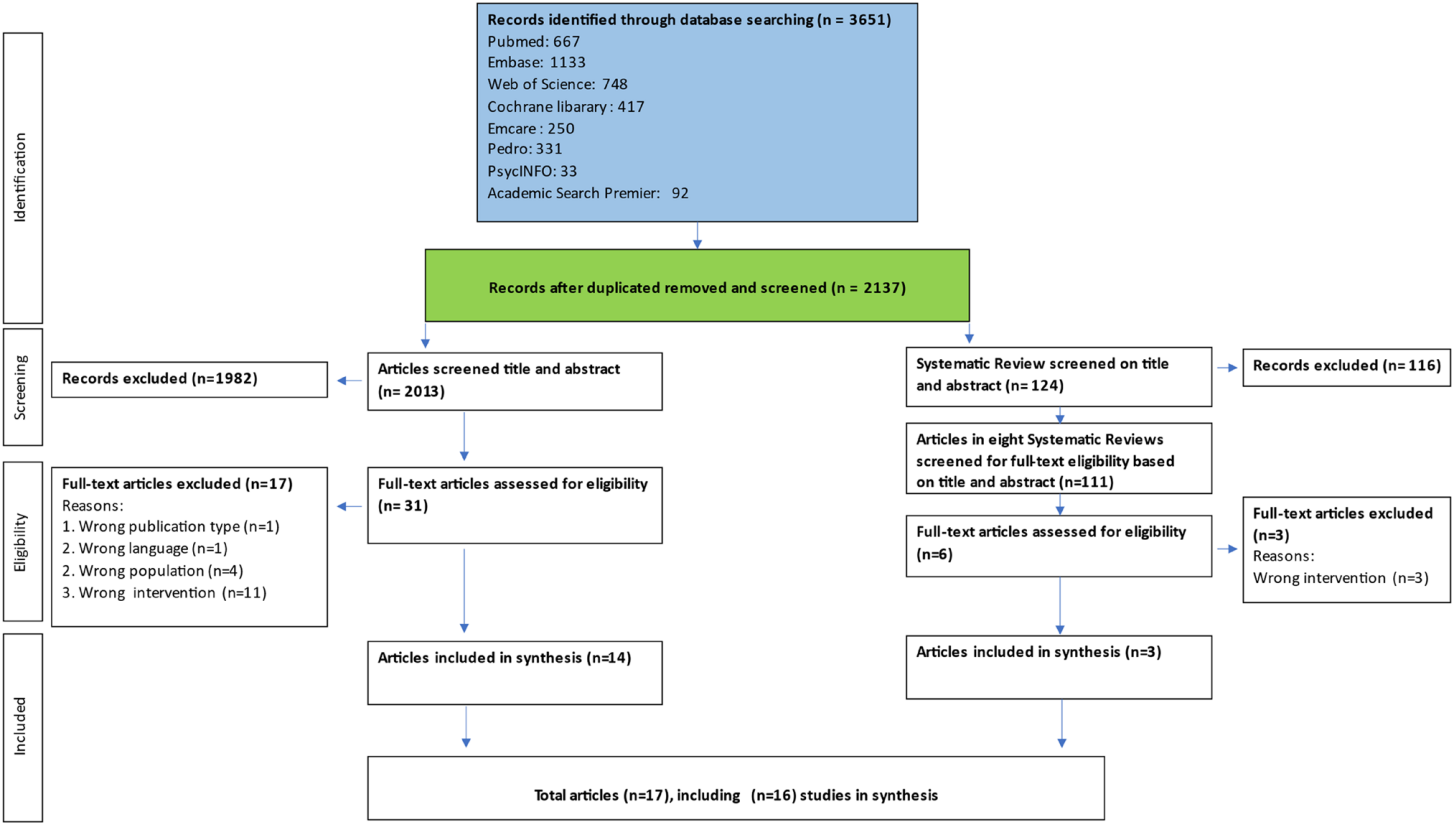
Preferred Reporting Items for Systematic Reviews and Meta-analyses (PRISMA): flow diagram of selected studies

After removing duplicates, the search yielded 2.137 records, of which 124 were systematic reviews. Titles and abstracts of the 2.013 non-systematic reviews were screened from which 31 records were selected for screening of the full text papers, with 14 of these meeting the eligibility criteria. Of the 124 systematic reviews, eight were considered relevant to the research question, and included 111 clinical studies. After removing duplicates or not meeting the eligibility criteria, six of those papers were selected for full-text review resulting in the inclusion of three articles. So, in total 17 articles, describing 16 studies, met the eligibility criteria and were included in this review (Fig. 1: Flow diagram of selected studies).

### General characteristics of included studies and populations

General characteristics of the included studies are shown in Table 1. The 16 studies included a total of 858 participants across seven countries (United States, Canada, Sweden, France, Jordan, Japan and Australia). Ten studies included patients with knee OA, hip OA or OA (not specified), with a mean age of the participants ranging from 40 to 74 years [37–47]. Three studies included patients who underwent an unilateral TKR or TKA, participants included in these studies had a mean age ranging from 60 to 68 years [48–50]. The other three studies included patients with RA [51], spondyloarthritis [52] and RA or Systemic Lupus Erythematosus (SLE) [53] with a mean age of 55 years [51], 52 years [52] and 55 years [53], respectively. All but three of 16 studies were RCTs [41, 44, 47]. The exceptions concerned a pre–post-study without a control group [47] and the other two were feasibility studies including two intervention groups [41, 44]. Four of the RCTs had a controlled delay group [38–40, 53] and the other nine studies a parallel control group. The duration of interventions ranged from 1 week to 6 months. In the 13 studies with a control group, the control group received education (*n* = 2) [50, 51], usual care (*n* = 1) [50], monthly or weekly phone calls to discuss overall health (*n* = 2) [48, 50], physical therapy/activity (*n* = 4) [37, 48, 49, 52], newsletter/email about their disease (*n* = 2) [38, 53], theoretical group session with information on OA (*n* = 1) [43], individual appointment with a physical therapist for specific exercises based on needs and goals (*n* = 1) [43], and/or a blinded WAT (*n* = 1) [45].

**Table 1:** General characteristics of the included studies

First author, year, country	Study design	Diagnosis	Inclusion and exclusion criteria	Subjects, n, mean age (SD), female (*n*)
Labat 2022, France [52]	RCT, 2 groups: E: Two weekly sessions of PA activity of their choice, wearing a Garmin Vivofit 4 with daily step goals and with weekly activity SMS reminders. After 24 weeks participants received an hour of coach-supervised PA per week, besides the personal two weekly sessions organized independently and WAT C: Two weekly sessions of PA activity of their choice. After 24 weeks participants received an hour of coach-supervised PA per week, besides the personal two weekly sessions organized independently	Spondyloarthritis	*Inclusion:* – People over 18 years of age – Understood the objectives and constraints of the study – Were diagnosed with spondyloarthritis according to the Assessment of Spondyloarthritis International Society criteria – Lived in Nice or the surrounding 20-km area – Were certified as having no contraindication perform physical activities such as swimming or Nordic walking *Exclusion:* – People who had coronary artery disease, moderate to severe heart failure, uncontrolled hypertension, myocarditis, pericarditis or endocarditis, lung disease, any contraindication to PA – Were unable to go to the activity venue – Were already undergoing supervised PA in a club or with a sports coach – Who were pregnant or breastfeeding Furthermore, the participants were also excluded if during the study they experienced serious adverse events, withdrew their consent, and conducted any protocol violation	E: *n* = 55, mean age = 52.3 (13.6) years, female *n* = 40 C: *n* = 53, mean age = 50.7 (14.0) years, female n = 36
Plumb Vilardage 2022, United States [44]	Randomized feasibility and acceptability pilot trial: E1: Study workbook, two 45 min telephone delivered treatment session, and a fitness tracker Garmin Vivofit 4 for 6 weeks E2: Usual care plus a fitness tracker Garmin Vivofit 4 with handout for 6 weeks	HOA/KOA	*Inclusion:* – Adults aged 65 or older – Diagnosis of OA in the knee and/or hip – English speaking – Ability to participate in telephone sessions – Ability to ambulate even if assisted by a cane or walker – Rating worst pain and pain interference within the last week as a 3 or greater out of 10 *Exclusion:* – Planned surgery (including joint replacement surgery) during the study duration that would affect or limit mobility for more than 3 weeks – Major surgery requiring limited mobility within the past 3 months – Myocardial infarction within the past 3 months – Fall(s) within the past 3 months that led to immediate medical treatment – Current enrollment in cardiac rehabilitation – Presence of a serious psychiatric condition – Reported or suspected moderate cognitive impairment – Indication by a medical provider that exercise should only be medically supervised – Presence of other unmanaged medical condition (e.g., hypertension, diabetes, asthma, neurodegenerative condition) that might lead to unsafe participation as outlined in the Physical Activity Readiness Questionnaire Plus subsequently verified by electronic medical record review and/or via communication with patients’ treating medical team	Total: *n *= 39, mean age 71.77 (5.189) years, female *n *= 33 E1: *n* = 19 E2: *n* = 20
Ostlind 2021, Sweden [43]	Clustered RCT, 2 groups E: Supported Osteoarthritis Self-Management Program and Fitbit Flex 2, for 12 weeks C: Supported Osteoarthritis Self-Management Program, for 12 weeks	HOA/KOA	*Inclusion:* – Hip or knee OA – Working ≥ 50% (20 h. /week) – Aged between 18 and 67 years – Being able to understand Swedish in speech and writing – Able to walk and participate in some form of exercise – Access to a smartphone, tablet or computer to use the Fitbit-app – Able to wear a WAT for 12 weeks *Exclusion:* Not reported	E (2021): *n* = 74, mean age = 56.9 (5.2) years, female *n* = 64
Christiansen 2020, United States [48]	RCT, 2 groups E: Outpatient PT, 6 months of wearing a Fitbit Zip, with weekly step goals and monthly phone call to promote PA C: Outpatient PT, 6 months of monthly phone call to discuss overall health	Unilateral TKR	*Inclusion:* – 45 years of age – Unilateral TKR – Self-reported “yes” when asked if they were interested in increasing PA *Exclusion:* – Any additional comorbidities that would prevent them from participating in a PA intervention – Another lower extremity surgery in the previous 6 months or had another lower extremity surgery planned within 6 months after enrolling in the study	E: *n* = 20, mean age = 66.5 (6.9) years, female *n* = 8 C: *n* = 23, mean age = 67.5 (7.2) years, female *n* = 15
Li 2020a, Canada [38]	RCT, delayed-control design, 2 groups E: Group education and individual counseling with a physical therapist, a Fitbit Flex 2 for 12 weeks and 4 bi-weekly calls over 8 weeks C: A monthly electronic newsletter of arthritis news in weeks 1 to 12 and started the same intervention in week 14	KOA	*Inclusion:* – Patients who had a physician-confirmed diagnosis of KOA or were aged ≥ 50 years and had felt pain or discomfort in or around the knee during the previous year lasting > 28 separate or consecutive days – Who had no previous diagnosis of RA, psoriatic arthritis, ankylosing spondylitis, polymyalgia rheumatica, connective tissue diseases, fibromyalgia, or gout – Who had no history of using disease-modifying antirheumatic drugs or gout medications – Who had no prior knee arthroplasty and not on a waiting list for total knee or hip replacement surgery – Who did not have surgery in the back, hip, knee, foot, or ankle joints in the past 12 months – Who had no history of acute injury to the knee in the past 6 months – Who had an email address and access to the internet daily – Who were able to attend a 1.5-h group education session *Exclusion:* – Patients who had previously used a physical activity wearable tracker – Who received a steroid injection in a knee in the last 6 months – Who received a hyaluronate injection in a knee in the last 6 months – Who used medication that may impair activity tolerance (e.g., beta blockers) – Who faced a level of risk by exercising as identified by the Physical Activity Readiness Questionnaire. If a participant did not pass the Physical Activity Readiness Questionnaire, a physician’s note was requested to determine the eligibility	E: *n* = 26, mean age = 65.0 (8) years, female *n* = 23 C: *n* = 25, mean age = 64.8 (9) years, female *n* = 19
Li 2020b, Canada [53]	RCT, delayed-control design, 2 groups E: Group education and individual counseling with a physical therapist, Fitbit Flex 2 and 4 bi-weekly follow-up calls over 8 weeks C: Monthly emails of arthritis news unrelated to physical activity. Started the same intervention in week 10	RA/SLE	*Inclusion:* – Physician-confirmed diagnosis of RA or SLE – An email address and daily access to the internet – Able to attend an in-person session *Exclusion:* – Used any physical activity wearable devices – Indicated that it was unsafe to be physically active without health professional supervision, as identified by the PAR-Q. If participants did not pass the PAR-Q, a physician’s note was required to determine eligibility	RA group: E: *n* = 43, mean age = 54.8 (15.4) years, female *n* = 38 C: *n* = 43, mean age = 55.3 (11.5) years, female *n* = 40 SLE group: E: *n* = 16, mean age = 49.9 (12.2) years, female *n* = 13 C: *n* = 16, mean age = 47.1 (13.8) years, female *n* = 14
Zaslavsky 2019, United States [47]	One group pre-posttest pilot study E: 14 weeks of wearing Fitbit Charge 2, weekly text messages, three phone calls motivational interviewing principles (week 1, 5 and 9)	OA	*Inclusion:* – Being age 65 years and older – Having a diagnosis of OA – Having a smartphone – Having physical activity levels below the U.S. Department of Health and Human Services recommended guidelines evaluated using the Rapid Assessment of Physical Activity scale – Having Insomnia Severity Index score ≥ 12 *Exclusion:* – Having an acute injury associated with hip or knee pain – Inability to stand up without assistance – Having a Memory Impairment Screen for Telephone score of < 4 – Having severe hearing or visual impairment an acute episode or change in the treatment of psychiatric problems within the past 3 months	E: *n* = 24, mean age = 71 (4) years, female *n* = 17
Li 2018, Canada [40]	RCT, delayed-control design, 2 groups E: Education, FitBit Flex and a bi-weekly telephone call for activity counseling for 2 months C: Received the same intervention 2 months later	KOA	*Inclusion:* – Physician-confirmed diagnosis of knee OA – Or passed 2 criteria for early OA: (1) being age 50 years or older, and (2) having experienced pain or discomfort in or around the knee during the previous year lasting 28 or more separate or consecutive days *Exclusion:* – Diagnosis of inflammatory arthritis, connective tissue diseases, fibromyalgia, or gout – Used disease-modifying antirheumatic drugs or gout medications – Knee arthroplasty – On a waitlist to receive knee or hip arthroplasty – Any surgery in the back, hip, knee, foot, or ankle joint in the past 12 months – Acute knee injury in the past 6 months – Received a steroid injection or hyaluronate injection in a knee in the last 6 months – BMI of 40 kg/m2 or higher – No email address or daily access to a personal computer with Internet access – Unable to attend the required education session in person – Using medications that impaired activity tolerance (e.g., beta-blockers); and had an inappropriate level of risk for increasing their unsupervised physical activity	E: *n* = 30, mean age = 61.3 (9.4) years, female *n* = 22 C: *n* = 30, mean age = 62.1 (8.5) years, female *n* = 28
Paxton 2018, United States [50]	RCT, 2 groups E: Daily physical activity goals, Fitbit Zip and weekly phone meetings for 12 weeks C: Usual care after TKA, weekly phone meetings to monitor participants’ health status for 12 weeks	Unilateral TKA	*Inclusion:* – 50–75 years – Underwent unilateral TKA *Exclusion:* Not reported	E: *n* = 22, mean age = 63 (7) years, female *n* = 13 C: *n* = 23, mean age = 64 (6) years, female *n* = 11
Darabseh 2017, Jordan [49]	RCT, 2 groups E: Pedometer Omron HJ-320 along with usual physiotherapy program for 7 days C: Physiotherapy program without pedometer	TKR	*Inclusion:* – TKR female patients – Osteoarthritis patients – Age group between 50–80 years old – Able to give informed consent – Able to return for follow-up *Exclusion:* – Bilateral TKR – Either hip or knee replacement in the last 12 months – Severe locomotor limitation due to cardio-respiratory dysfunction, central or peripheral nervous system deficits, spinal conditions, and other musculoskeletal disabilities	Total: *n* = 20, mean age = 63.38 (6.76) years E: *n* = 10, mean age = 60.09 (5.13) years, female *n* = 10 C: *n* = 10, mean age = 62.00 (6.66) years, female *n* = 10
Katz 2017, United States [51]	RCT, 3 groups E1: Education, Fitbit Flex for 20 weeks and step-monitoring diary E2: Education, Fitbit Flex for 20 weeks and step-monitoring diary plus step targets C: Education	RA	*Inclusion:* – Physician-diagnosed RA – English- or Spanish-speaking – Able to attend 3 in-person research visits – Presence of greater than minimal fatigue *Exclusion:* – BMI < 20 kg /m2 – Currently engaging in regular exercise, and non-ambulatory or presence of a condition that would limit the ability to walk (e.g., foot deformities, lower-extremity joint surgery upcoming or in past 6 months, myocardial infarction in past 6 months, stroke, congestive heart failure, or severe chronic obstructive pulmonary disease)	E1: *n* = 34, mean age = 55.9 (12.4) years, female *n* = 30 E2: *n* = 34, mean age = 50.2 (14.1) years, female *n* = 30 C: *n* = 28, mean age = 59.1 (12.5) years, female *n* = 24
Li 2017, Canada [39]	RCT, delayed-control design, 2 groups E: Education, Fitbit Flex and a weekly telephone call for activity counseling for 1 month C: Received the same intervention 1 month later	KOA	*Inclusion:* – Physician-confirmed diagnosis of KOA – Or passed 2 criteria for early OA: (1) being age 50 years or older, and (2) having experienced pain or discomfort in or around the knee during the previous year lasting 28 or more separate or consecutive days *Exclusion:* – Diagnosis of inflammatory arthritis, connective tissue diseases, fibromyalgia or gout – Used disease-modifying antirheumatic drugs or gout medications – Knee arthroplasty – On the waitlist to receive total knee arthroplasty – Acute knee injury in the past 6 months – No email address or daily access to a personal computer with Internet access – BMI of 40 kg/m2 or more – Received a steroid injection in the last 6 months – Received hyaluronate injection in a knee in the last 6 months using medications that impaired activity tolerance (such as β-blockers) or had an inappropriate level of risk for increasing their unsupervised physical activity	E: *n* = 17, mean age = 52.3 (9.7) years, female *n* = 14 C: *n* = 17, mean age = 58.7 (6) years, female *n* = 14
Skrepnik 2017, United States [45]	RCT, 2 groups E: 6-mL injection of hylan G-F 20, brochure from the Arthritis Foundation, unblinded Jawbune UP 24 and OA GO app for 90 days C: 6-mL injection of hylan G-F 20, blinded Jawbune UP 24 for 90 days	KOA	*Inclusion:* – Unilateral KOA – Have been suitable for treatment with hylan G-F 20 based on the decision of the physician investigator *Exclusion:* – Aged younger than 30 years or older than 80 years – Were unfamiliar with smartphones – Or had baseline pain greater than 9 on the 11-point NPRS in the target-for-treatment knee while walking on a flat surface – Patients with bilateral disease were excluded with the exception of patients who were treated in only one knee and had contralateral knee pain less than 4 on NPRS while walking on a flat surface – Patients whose baseline daily step average was less than 500 or more than 8000 as assessed during the screening and run-in phases were not eligible – A BMI greater than 35 or life expectancy less than 12 month – Were currently using a wearable activity monitor or analogous device – Had planned surgery on any lower extremity joint or any significant medical condition that would interfere with study participation – Were chronic narcotic users – Pregnant or breastfeeding or likely to become pregnant	E: *n* = 107, mean age = 61.6 (9.5) years, female *n* = 59 C: *n* = 104, mean age = 63.6 (9.3) years, female *n* = 47
Hiyama 2011, Japan [37]	RCT, 2 groups E: 4 weeks of wearing a pedometer and ice therapy, range of motion exercises, muscle strengthening C: 4 weeks of ice therapy, range of motion exercises, muscle strengthening	KOA	*Inclusion:* – KOA *Exclusion:* – Progressive or debilitating – Conditions (metastatic cancer, major stroke or crippling arthritis) that would limit participation in a walking protocol – Pre-existing total knee arthritis or meniscectomy; or other musculoskeletal system disorders or secondary osteoarthritis of other joints – Rest pain and difficulty increasing the number of steps walked daily; or cognitive impairment as measured by a score on the MMSE < 24 points	E: *n* = 20, mean age = 71.9 (5.2) years, female *n* = 20 C: *n* = 20, mean age = 73.8 (5.7) years, female *n* = 20
Ng 2010, Australia [41]	Randomized feasibility trial, 2 groups E1: Walking program 3 days a week for 12 weeks, pedometer use for 12 weeks and glucosamine sulphate per day for 6 weeks E2: Walking program 5 days a week for 12 weeks, pedometer use for 12 weeks and glucosamine sulphate per day for 6 weeks	HOA/KOA	*Inclusion:* – Aged 40 to 75 years – Physician-diagnosed OA in at least one hip or knee (verified by a doctor’s letter confirming diagnosis) – Experiencing pain, stiffness, crepitus and difficulty with daily activities within the previous month – Ability to walk at least 15 min continuously – Ability to safely participate in moderate-intensity exercise, as determined by the Sports Medicine Australia Stage I pre-exercise screening questions *Exclusion:* – Other forms of arthritis; – Corticosteroid or viscosupplement injections within the previous three months – History of infection in a knee or hip – Living in a dependent environment – Taking daily medication for OA, including analgesia; or were allergic to shellfish – Individuals who were planning to have surgery in the next six months – Receiving psychiatric or psychological treatment – Pregnant or planning to become pregnant – Exercising more than 60 min per week – Participating in another research study	E1: *n* = 15, mean age = 40–59 (n = 7) 60–75 (*n* = 8) years, female *n* = 10 E2: *n* = 13, mean age = 40–59 (*n* = 4) 60–75 (*n* = 9) years, female *n* = 7
Talbot 2003, United States [46]	RCT, 2 groups E: Arthritis Self-Management program and pedometer for 12 weeks C: Arthritis Self-Management program	KOA	*Inclusion:* – Aged 60 and older – Pain in one or both knees on most days – Difficulty performing at least one functional task because of pain and radiographic evidence of OA *Exclusion:* – Current participation in an exercise research study – A medical condition for which exercise is contraindicated, such as unstable angina pectoris or recent myocardial infarction a score of less than 24 on the MMSE	E: *n* = 17, mean age = 69.6 (6.7) years, female *n* = 13 C: *n* = 17, mean age = 70.7 (4.7) years, female *n* = 13

*RCT* randomized controlled trial, *E* experimental group, *C* control group, *N* number, *SD* standard deviation, *OA* osteoarthritis, *KOA* knee osteoarthritis, *HOA* hip osteoarthritis, *RA* rheumatoid arthritis, *SLE* systemic lupus erythematosus, *TKR* total knee replacement, *TKA* total knee arthroplasty, *PA* physical activity, *PT* physical therapy, *WAT* wearable activity tracker, *PAR*-*Q* physical activity readiness questionnaire, *MMSE* mini-mental state examination, *BMI* body mass index, *NPRS* numeric pain rating scale

### Characteristics of the WAT

Detailed description on the type and brand of equipment (WAT) was reported in 14 of 16 studies (88%). Nine studies used the Fitbit®, with some variation regarding the type. In five of the studies a Fitbit Flex® was used [38–40, 43, 53], in three studies the Fitbit Zip® [48, 50, 51] and in one study the FitBit Charge 2® [47]. It was reported that the Fitbit Zip® was worn around the waist [48], whereas the Fitbit Flex (2)® and Fitbit Charge 2® were worn around the wrist [38–40, 43, 47]. Three of these nine studies did not explain the location of wearing of the Fitbit Flex or Zip® [50, 51, 53]. Two studies used a Garmin Vivofit 4.0®, worn around the wrist [44, 52]. Three other studies used an electronic pedometer as WAT (Jawbone UP 24® [45] worn around the waist, KenzLifecoder EX® worn around the wrist [37] and Omron HJ-320® [49] location of wearing unknown). Two studies did not report the type and brand of the WAT at all [41, 46]. Characteristic of the WATs are described in Online Resource 3.

### Determining the starting level and tailoring of the use of a WAT in the PA program

Description of a decision rule to determine the starting level at which people start the PA program with a WAT was reported in 31% of the studies (5/16 studies) [37, 45–47, 51]. The starting level of the (step) goal in these five studies was based on the average daily steps of the first week. The other 11 studies did not describe any information on the starting level. Description of the PA programs/exercises and if they were generic or tailored are described in all but one of the studies (94%) [49], but a detailed description of how exercise were tailored to the individual was only described by 25% of the studies (4/16 studies) [47, 50, 51, 53]. Seven of the 16 studies (44%) described how the progression of the PA program was executed [37, 41, 46–48, 50, 51]; however, the decision rule(s) for determining exercise progression was only described by 13% of the studies (2/16 studies) [47, 50]. Details are also described in Online Resource 3.

### Non-exercise components or motivational strategies in PA programs

All but one of the studies reported non-exercise components or motivational strategies [49]. In six of the 16 studies instructions on the use of the WAT was included [43–45, 47, 48, 50]. Another non-exercise component was the information and/or education given about PA and/or self-management of the disease. This information/education was given in group sessions by nine of the 16 studies [38–40, 43, 46, 50–53] and/or in newsletters, guides or booklets by six of the 16 studies [41, 44–46, 50, 51]. Motivational strategies were also included in ten of the 16 studies, including personal counseling in PA goals. In seven of those ten studies, weekly or bi-weekly phone calls were made to monitor and/or recall the PA goals [38–40, 43, 46, 47, 53], three studies did this by text messages [45, 47, 52]. Details on the non-exercise components and motivational strategies given during the PA intervention are stated in Online Resource 4.

### Adherence, fidelity and adverse events

In nine of the 16 studies, the measurement of adherence to the intervention was described [38, 39, 43, 45, 46, 48, 50, 51, 53]. The adherence measured by attendance to education sessions [38, 46, 53], use of the WAT [38, 39, 43, 46, 48, 50, 51, 53], and participation in PA goal counselling sessions [38, 53]. Of the 16 included studies, seven studies reported information about the type and number of adverse events [38–40, 45, 48, 51, 53]. Details on the adherence, fidelity and adverse events are stated in Online Resource 5.

### Risk of bias assessment

Full consensus was reached between researchers MB and MVW on risk of bias assessment. Overall, the methodological quality of the included trails was moderate to good. Six of the sixteen studies had a low [37, 38, 43, 45, 46, 53] and ten studie had a moderate risk of bias [39–41, 44, 47–52], whereas the studies from Paxton et al. and Darabseh et al. had the highest risk of bias [49, 50]. The randomization process was not clearly described in three of the studies [49–51]. Also, the selection of the reported results were not clearly described for four of the studies [40, 48, 49, 52]. Details are described in Online Resources 6 and 7.

### Statistical analyses and synthesis

None of the studies had a complete reporting of the process of delivery of interventions using WATs to increase PA in patients with RMDs. For all studies the reporting quality was poor to moderate, according to both the CERT and CONSORT E-Health checklists. According to the CERT checklist, the reporting quality was classified as poor for six of the 16 studies [37, 43–45, 49, 52] and moderate for the other ten studies [38–41, 46–48, 50, 51, 53]. Of the ten studies with moderate reporting quality, the two studies with the highest quality had reported 68% of the items adequately [47, 48], the other eight studies showed lower percentages [38–41, 46, 50, 51, 53].

According to the CONSORT E-Health checklist the reporting quality was classified as poor for eight studies [37, 41, 45–47, 49–51], and the other eight of the 16 studies had a moderate reporting quality [38–40, 43, 44, 48, 52, 53]. All eight moderate reporting quality studies scored 58% of the items adequately. An overview of the reporting quality of the studies is shown in Table 2.

**Table 2:**
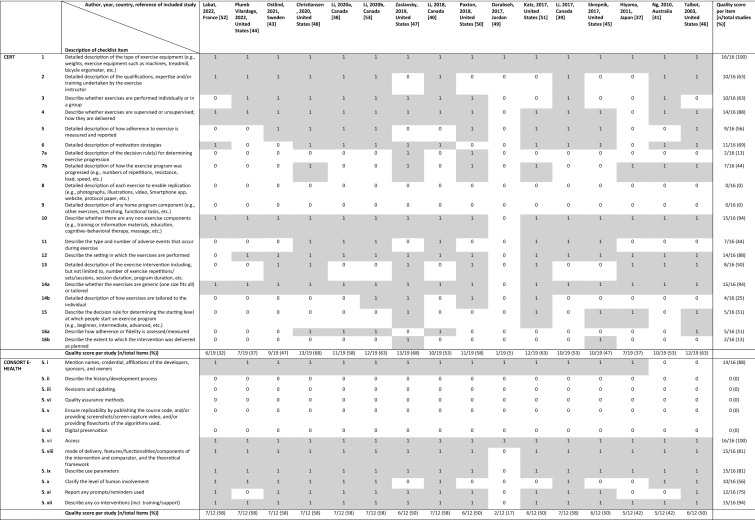
Reporting quality of included studies according to the CERT and CONSORT E-Health checklist

0: Item is not or not clearly reported, 1: Item is reported, n: number

Of the CERT checklist, two of the 19 items were not reported in any of the studies (i.e. description of each exercise to enable replication and any home program components) and 8 of the 19 items were only reported by 50% or less of the studies (i.e. description of: the decision rule(s) for determining exercise progression; how the exercise program was progressed; the type and number of adverse events that occur during exercise; the exercise intervention including, but not limited to, number of exercise repetitions/sets/sessions, session duration, program duration; how exercises are tailored to the individual; the decision rule for determining the starting level at which people start an exercise program; how adherence or fidelity is assessed/measured; the extent to which the intervention was delivered as planned). Four of the 19 CERT items were reported by 51–79% of the studies (i.e. description of: the qualifications, expertise and/or training undertaken by the exercise instructor; motivational strategies; of how adherence to exercise is measured and reported; exercises are performed individually or in a group), and five of the 19 items were reported in more than 80% of the studies (i.e. description of: the type of exercise equipment; whether the exercises are generic (one size fits all) or tailored; exercises are supervised or unsupervised, how they are delivered; the setting in which the exercises are performed; whether there are any non-exercise components). In total five of the 12 CONSORT E-Health checklist items were not reported by any of the studies; description of the history and development process, revisions and updating, quality assurance methods and digital preservation. Two items of the CONSORT E-Health checklist were reported in 51–79% of the studies (i.e. description of the level of human involvement and any prompts/reminders used) and five items were reported in 80% or more of the studies (i.e. description of: developers/owners; access; mode of delivery of the intervention; use parameters and any co-interventions). An overview of the reporting quality of the checklist items are also shown in Table 2.

There was only limited overlap between the studies with poor reporting quality and those with a high risk of bias. Of the ten studies with a moderate risk of bias, three also showed poor reporting quality on the CERT checklist [47, 49, 52] and four [41, 49–51] on the CONSORT E-Health checklist.

### Discussion

In this systematic review on the reporting quality of interventions to increase PA in patients with RMDs using WATs, it was found that overall the reporting quality was moderate to poor. Based on two checklists, for the reporting of exercise interventions (CERT) and eHealth interventions (CONSORT E-Health), the best reported items concerned the description of the equipment, supervision of the intervention and whether there were any non-exercise components included in the intervention. On the other hand, information on the description of the starting level, decision rules and progression of exercises, the description and tailoring of the exercises, adverse events, fidelity of the intervention, revisions and update of WATs and accessory quality assurance methods were in general not or poorly reported and are points of improvement for future studies.

Moderate to poor reporting quality of interventions targeting PA appears to be a more common problem. A previous systematic review on the effect of WATs on levels of PA in patients with various (chronic) diseases including RMDs concluded that the description of the interventions was heterogenous [16]. To date, no studies have systematically assessed the reporting quality of PA interventions including a WAT in patients with RMDs. Recently published systematic reviews on the reporting quality of PA promotion concern interventions without the use of a WAT in patients with low back pain [26], hip OA [25], pulmonary hypertension [54], progressive supranuclear palsy [55] and JIA [28] are in line with the results of our study, and concluded that the reporting quality of PA promotion interventions (all using the CERT checklist) was generally moderate to low. However, the findings on specific CERT items that were not or poorly reported were mixed and sometimes even contradictory. In addition, there is only one systematic review on the reporting quality of digital interventions using the CONSORT E-Health checklist, regarding digital interventions in general, in patients with cardiometabolic conditions [56]. That review used eight of the CONSORT E-Health items, and also concluded an overall inconsistent reporting of the interventions. Their finding regarding insufficient reporting of the development process is in line with the results of our study. Moreover, the results of previous studies and our study underline the need to develop better guidelines for the reporting of interventions targeting PA using WATs.

Complete reporting may have been limited due to lack of requirements from journals for authors to use the appropriate reporting guidelines, or journal restrictions on e.g. the length of a manuscript or number of tables. This was confirmed in a systematic review of Abell et al. where the completeness of reporting on exercise-based interventions increased from 8 to 43% after additional information was requested by corresponding authors [57]. In contrast to that study, no additional information was requested from authors within our systematic review. In case of incomplete reporting of specific elements, only the available study protocols or websites were consulted. Thus, it cannot be ruled out that failure to consult the corresponding author may have led to an underestimation of the reporting quality of the included studies. The CERT [22] and CONSORT E-Health checklist [24] were already published in 2016 and 2011. All but three studies in this review [37, 41, 46] were published after the checklists were available. The impact of these checklists on the reporting quality therefore appears to be limited.

A complicating factor in assessing the reporting quality of the delivery of WATs is the suitability of currently available sets of criteria for doing so. First of all, the CERT and CONSORT E-Health were designed as reporting guidelines and not as a measure to assess the reporting quality. To our knowledge, such reporting quality assessment instruments are not available. In this systematic review both reporting quality checklists were used, as both comprised elements that are relevant for the delivery of WATs to promote PA. Although there was some overlap regarding their contents, the description of items varied considerably, and both also comprised elements that were unique. Overall, neither of the checklists nor their combination appeared to be complete. As WATs are more and more used in PA promotion, the development of sets of criteria to report the delivery process of WATs and assess the reporting quality is needed. These sets could also consist of items already present in existing CERT and CONSORT E-Health checklists. While not within the scope of this systematic review, it would be interesting to assess the effect of the PA interventions using a WAT. However, no effects could be measured due to the heterogenous and moderate to poor reporting quality of the interventions. Future research, including more studies in RMD patients and with better reporting quality, should discuss the effect of PA interventions including a WAT in RMD patients.

This overview of reporting quality and the subsequent results may have implications for researchers and clinicians. For researchers, improvement of the reporting quality will increase the accurate application of WATs in clinical trials, make it easier to replicate studies and compare their results. Only when interventions are described in a comprehensive and standardized manner data from different studies can be analyzed and pooled. To improve the reporting quality, researchers may use the combination of the CERT [24] and CONSORT E-Health [22] checklists. However, the current systematic review demonstrated that the combination of both resulted in incomplete reporting of some aspects that are particularly relevant for WATs. Thus, the development of a specific guideline for reporting of interventions aimed at PA promotion with the use of a WAT is desired. For clinicians, improved reporting quality will increase the therapeutic validity of the interventions they are offering. Currently, the lack of relevant and/or detailed information hinders clinicians from implementing and carrying out the intervention as intended, because, e.g. information about determining the starting level and decision rules for progression or tailoring of PA using a WAT is lacking. Until now, clinicians may have to contact the corresponding authors for additional information on the methods used, which is not considered to be feasible for clinical practice.

A strength of the current study is the comprehensive evaluation of the reporting quality using two existing checklists and highlighting the key items for improvement in the reporting of interventions using WATs to increase PA. A systematic search was completed in most major databases, with a complementary hand search of systematic reviews to ensure that no relevant studies were missed. A limitation is that a larger number of studies would allow for sub-analysis of other characteristics of the studies, such as the publication journal and its author guidelines. Moreover, this systematic review included only three studies with patients with IA [51–53]. The observed difference between the number of identified studies on IA as compared to OA is likely to be related to the difference in the prevalence of OA and IA, with hip and knee OA being far more prevalent than IA. Likewise, the number of clinical trials on exercise and PA promotion is much more extensive in OA than in IA. Nevertheless, it is likely that the principles of the delivery of PA interventions with the use of a WAT are similar in OA and IA, and there is little ground for the expectation that the reporting quality of PA promotion interventions using a WAT would be different. So, although exceptions cannot be ruled out, it is likely that the conclusions of this review on the reporting quality are generalizable to studies on PA promotion for different RMDs. Regarding the practical approach to PA promotion in patients with different conditions, it is beyond doubt that this may differ at the individual patient level, in part due to differences in the clinical features of their underlying condition. However, the basic principles of exercise and PA promotion are similar, as is, e.g. reflected in the 2018 EULAR (European Alliance of Associations for Rheumatology) recommendations for PA for people with IA and OA [58] that explicitly address both IA and OA together.

### Conclusion

This study provides a first overview of the reporting quality of interventions using WATs to increase PA in patients with RMDs. While PA interventions using WATs have the potential to benefit people with RMDs [17], the moderate to poor reporting quality of PA interventions using WATs limits future replication and assessment of effects. The development of criteria to report on the use of WATs for PA promotion is needed and can improve the reporting quality and clinical usefulness of future studies.

## Supplementary Information

Below is the link to the electronic supplementary material.

Supplementary file1 (DOCX 19 KB)

Supplementary file2 (DOCX 18 KB)

Supplementary file3 (DOCX 42 KB)

Supplementary file4 (DOCX 45 KB)

Supplementary file5 (DOCX 36 KB)

Supplementary file6 (DOCX 123 KB)

Supplementary file7 (DOCX 49 KB)

## Data Availability

The datasets generated during and/or analysed during the current study are available from the corresponding author on reasonable request.
