# Supplementary material for: Reporting quality of interventions using a wearable activity tracker to improve physical activity in patients with inflammatory arthritis or osteoarthritis: a systematic review
Source: Rheumatol Int. 2022 Dec 1;43(5):803–24. doi: 10.1007/s00296-022-05241-x (PMC10073167; doi:10.1007/s00296-022-05241-x)
Supplement: Supplementary file 1 — Supplementary file1 (DOCX 19 KB) [file 296_2022_5241_MOESM1_ESM.docx]

Article title: Reporting quality of interventions using a wearable activity tracker to improve physical activity in patients with inflammatory arthritis or osteoarthritis: a systematic review

Journal: Rheumatology International

M.A.T. van Wissen^1^*, M.A.M. Berger^2^, J.W. Schoones^3^, M.G.J. Gademan^1, 4^, C.H.M. van den Ende^5,6^, T.P.M. Vliet Vlieland^1^, S.F.E. van Weely^1^

1.Department of Orthopaedics, Rehabilitation and Physical Therapy, Leiden University Medical Center, Leiden, The Netherlands; 2.The Hague University of applied sciences, The Hague, The Netherlands; 3. Directorate of Research Policy (Walaeus Library), Leiden, The Netherlands;4. Department of Clinical Epidemiology, Leiden University Medical Center, Leiden, The Netherlands; 5. Department of Research, Sint Maartenskliniek, Nijmegen, The Netherlands; 6.Department of Rheumatology, Radboud University Medical Center, Nijmegen, The Netherlands

*Corresponding author: M.A.T. van Wissen. m.a.t.van_wissen@lumc.nl

# **Supplementary Table S1 Search strategy**

| P (population) | People with RA, OA (lower extremities), AxSpa, psoriatic arthritis or juvenile arthritis (RMD’s). |
| --- | --- |
| I (intervention) | Physical Activity promotion with use of wearable activity tracker/pedometer. Exercise promotion with use of wearable activity tracker/pedometer . |
| C (comparison) | Usual care, attention to control, no treatment, waiting list control or physical activity promotion without use of wearable activity tracker/pedometer. |
| O (outcome) | 1. Characteristics of the study protocol/intervention recording to promotion of physical activity Characteristics of the intervention related to the wearable activity tracker. 2. Adherence to the intervention and intervention related to the wearable activity tracer 3. Barriers and facilitators regarding to intervention and intervention related to the wearable activity tracker |
| Study types | Systematic literature reviews, meta-analyses, RCTs, qualitative studies, study designs, pilot studies and observational studies |

**Search strategy (PubMed):****(**(("**Arthritis, Rheumatoid**"[mesh] OR "rheumatoid arthritis"[tw] OR "inflammatory arthritis"[tw] OR "**Osteoarthritis**"[mesh] OR "osteoarthritis"[tw] OR osteoarthrit*[tw] OR "osteoarthrosis"[tw] OR osteoarthro*[tw] OR "degenerative arthritis"[tw] OR degenerative arthriti*[tw] OR "osteoarthrosis deformans"[tw] OR coxarthro*[tw] OR gonarthro*[tw] OR **"Spondylarthropathies"[mesh] OR "spondylarthropathies"[tw] OR "spondylarthropathy"[tw] OR spondylarthropath*[tw]** OR "Marie-Strumpell Spondylitis"[tw] OR Bechterew*[tw] OR "Spondylitis, Ankylosing"[mesh] OR "ankylosing spondylitis"[tw] OR "Spondyloarthritis Ankylopoietica"[tw] OR "Ankylosing Spondylarthritis"[tw] OR "Spondylarthritis Ankylopoietica"[tw] OR Bechterew*[tw] OR "Marie-Struempell Disease"[tw] OR "Marie Struempell Disease"[tw] OR "Rheumatoid Spondylitis"[tw] OR "Spondylitis Ankylopoietica"[tw] OR "Ankylosing Spondyloarthritis"[tw] OR "Spondylarthritis"[mesh] OR spondyloarthrit*[tw] OR spondyloartrit*[tw] OR "spondylo-arthrit*"[tw] OR "Arthritis, Reactive"[mesh] OR "Reactive Arthritis"[tw] OR "Post-Infectious Arthritis"[tw] OR "Postinfectious Arthritis"[tw] OR "Reiter Syndrome"[tw] OR "Reiter's Disease"[tw] OR "Reiters Disease"[tw] OR "Reiter Disease"[tw] OR **"Arthritis, Psoriatic"[mesh]** OR "Arthritic Psoriasis"[tw] OR "Psoriatic Arthritis"[tw] OR "Psoriasis Arthropathica"[tw] OR "Psoriatic Arthropathy"[tw] OR "Psoriatic Arthropathies"[tw] OR **"Arthritis, Juvenile"[Mesh]** OR "juvenile idiopathic arthritis"[tw] OR "Juvenile Arthritis"[tw] OR "Juvenile Chronic Arthritis"[tw] OR "Juvenile Enthesitis-Related Arthritis"[tw] OR "Juvenile Idiopathic Arthritis"[tw] OR "Juvenile Oligoarthritis"[tw] OR "Juvenile Psoriatic Arthritis"[tw] OR "Juvenile Rheumatoid Arthritis"[tw] OR "Juvenile Systemic Arthritis"[tw] OR "Juvenile-Onset Still's Disease"[tw]) AND **("Exercise Therapy"[mesh] OR "exercise therapy"[tw] OR exercise therap*[tw] OR "Muscle Stretching Exercises"[tw] OR "Muscle Stretching Exercise"[tw] OR "Static Stretching"[tw] OR "Passive Stretching"[tw] OR "Static-Passive Stretching"[tw] OR "Static Passive Stretching"[tw] OR "Isometric Stretching"[tw] OR "Active Stretching"[tw] OR "Static-Active Stretching"[tw] OR "Static Active Stretching"[tw] OR "Ballistic Stretching"[tw] OR "Dynamic Stretching"[tw] OR "PNF Stretching"[tw] OR "Plyometric Exercise"[tw] OR "Plyometric Exercises"[tw] OR Plyometric Drill*[tw] OR "Plyometric Drills"[tw] OR "Plyometric Training"[tw] OR "Plyometric Trainings"[tw] OR "Stretch-Shortening Exercise"[tw] OR "Stretch Shortening Exercise"[tw] OR "Stretch-Shortening Exercises"[tw] OR "Stretch-Shortening"[tw] OR "Stretch Shortening"[tw] OR "Stretch-Shortening Drills"[tw] OR "Stretch-Shortening Cycle Exercise"[tw] OR "Stretch Shortening Cycle Exercise"[tw] OR "Stretch-Shortening Cycle Exercises"[tw] OR "Resistance Training"[tw] OR "Strength Training"[tw] OR "training"[tw] OR "Weight-Lifting"[tw] OR "Weight Lifting"[tw] OR "Weight-Bearing"[tw] OR "Weight Bearing"[tw] OR "Exercise"[mesh] OR exercis*[tw] OR "stretching"[tw] OR "Exercise"[tw] OR "Exercises"[tw] OR "Physical Exercise"[tw] OR "Physical Exercises"[tw] OR "Isometric Exercises"[tw] OR "Isometric Exercise"[tw] OR "Aerobic Exercises"[tw] OR "Aerobic Exercise"[tw] OR "Circuit-Based Exercise"[tw] OR "Cool-Down Exercise"[tw] OR "Cool-Down Exercises"[tw] OR "Physical Conditioning"[tw] OR "Running"[tw] OR "Jogging"[tw] OR "Swimming"[tw] OR "Walking"[tw] OR "Warm-Up Exercise"[tw] OR "Warm-Up Exercises"[tw] OR "Physical Exertion"[mesh] OR "Physical Exertion"[tw] OR "Physical Effort"[tw] OR "Physical Efforts"[tw] OR "Physical Fitness"[mesh] OR "Physical Fitness"[tw] OR "Fitness"[tw] OR "Physical Endurance"[mesh] OR "Physical Endurance"[tw] OR "Anaerobic Threshold"[tw] OR "Exercise Tolerance"[tw] OR "Exercise Movement Techniques"[mesh] OR "Exercise Movement"[tw] OR "Bicycling"[tw] OR "Walking"[tw] OR "Motor Activity"[mesh] OR "Physical Activity"[tw] OR exertion*[tw] OR "Sports"[mesh] OR "sports"[tw] OR "sport"[tw] OR "Athletic Performance"[tw] OR "Cardiorespiratory Fitness"[tw] OR "Physical Endurance"[tw] OR "Physical Fitness"[tw] OR "Bicycling"[tw] OR "Golf"[tw] OR "Gymnastics"[tw] OR "Mountaineering"[tw] OR "Racquet Sports"[tw] OR "Tennis"[tw] OR "Running"[tw] OR "Jogging"[tw] OR "Skating"[tw] OR "Snow Sports"[tw] OR "Skiing"[tw] OR "Swimming"[tw] OR "Track and Field"[tw] OR "Volleyball"[tw] OR "Walking"[tw] OR "Weight Lifting"[tw] OR treadmill*[tw] OR row[tw] OR rows[tw] OR rowing[tw] OR muscle strength*[tw] OR "Range of Motion, Articular"[mesh] OR "Joint Range of Motion"[tw] OR "Joint Flexibility"[tw] OR "Range of Motion"[tw] OR "Postural Balance"[mesh]** OR **"Postural Balance"[tw])** AND ("Fitness Trackers"[Mesh] OR "Fitness Trackers"[tw] OR "Fitness Tracker"[tw] OR "Fitness Tracking"[tw] OR "wearable activity tracker"[tw] OR "wearable activity trackers"[tw] OR "wearable activity tracking"[tw] OR "activity tracker"[tw] OR "activity trackers"[tw] OR "activity tracking"[tw] OR "pedometer"[tw] OR "pedometers"[tw] OR pedomet*[tw] OR "Wearable Electronic Devices"[Mesh:NoExp] OR "wearables"[tw] OR "wearable"[tw] OR wearab*[tw] OR "electronic skin"[tw] OR "Monitoring, Ambulatory"[Mesh:NoExp] OR "Ambulatory Monitoring"[tw] OR "Fitbit Flex"[tw] OR "Smartwatch"[tw] OR "Smartwatches"[tw] OR "Smart watch"[tw] OR "Smart watches"[tw] OR "Actigraphy"[Mesh] OR "Actigraphy"[tw] OR Actigra*[tw] OR "tracker"[tw] OR "trackers"[tw] OR "step counter"[tw] OR "step counters"[tw] OR stepcount*[tw] OR "step count*"[tw] OR "Digi-Walker"[tw] OR "Walk4Life"[tw] OR "TrekLINQ"[tw] OR "App"[tw] OR "Apps"[tw] OR "FitBit"[tw] OR "Garmin"[tw] OR "Misfit"[tw] OR "Omron"[tw] OR "Apple Watch"[tw] OR "Apple Watches"[tw] OR "Mobile Applications"[Mesh] OR "Mobile Applications"[tw] OR "Mobile Application"[tw] OR "Portable Electronic Application"[tw] OR "Portable Electronic Applications"[tw] OR "Portable Software Application"[tw] OR "Portable Software Applications"[tw] OR (("Accelerometry"[mesh] OR accelero*[tw] OR monitor*[tw]) AND ("Cell Phone"[Mesh:NoExp] OR "cell phone"[tw] OR "cell phone"[tw] OR "smartphone"[tw] OR "smartphones"[tw] OR "smart phone"[tw] OR "smart phones"[tw] OR "telephone"[tw] OR "telephones"[tw] OR "mobile phone"[tw] OR "mobile phones"[tw] OR "gps"[tw] OR "global positioning systems"[tw] OR "global positioning system"[tw]))) AND ("2005/01/01"[PDAT] : "3000/12/31"[PDAT])) **OR** (("**Arthritis, Rheumatoid**"[majr] OR "rheumatoid arthritis"[ti] OR "inflammatory arthritis"[ti] OR "**Osteoarthritis**"[majr] OR "osteoarthritis"[ti] OR osteoarthrit*[ti] OR "osteoarthrosis"[ti] OR osteoarthro*[ti] OR "degenerative arthritis"[ti] OR degenerative arthriti*[ti] OR "osteoarthrosis deformans"[ti] OR coxarthro*[ti] OR gonarthro*[ti] OR **"Spondylarthropathies"[majr] OR "spondylarthropathies"[ti] OR "spondylarthropathy"[ti] OR spondylarthropath*[ti]** OR "Marie-Strumpell Spondylitis"[ti] OR Bechterew*[ti] OR "Spondylitis, Ankylosing"[majr] OR "ankylosing spondylitis"[ti] OR "Spondyloarthritis Ankylopoietica"[ti] OR "Ankylosing Spondylarthritis"[ti] OR "Spondylarthritis Ankylopoietica"[ti] OR Bechterew*[ti] OR "Marie-Struempell Disease"[ti] OR "Marie Struempell Disease"[ti] OR "Rheumatoid Spondylitis"[ti] OR "Spondylitis Ankylopoietica"[ti] OR "Ankylosing Spondyloarthritis"[ti] OR "Spondylarthritis"[majr] OR spondyloarthrit*[ti] OR spondyloartrit*[ti] OR "spondylo-arthrit*"[ti] OR "Arthritis, Reactive"[majr] OR "Reactive Arthritis"[ti] OR "Post-Infectious Arthritis"[ti] OR "Postinfectious Arthritis"[ti] OR "Reiter Syndrome"[ti] OR "Reiter's Disease"[ti] OR "Reiters Disease"[ti] OR "Reiter Disease"[ti] OR **"Arthritis, Psoriatic"[majr]** OR "Arthritic Psoriasis"[ti] OR "Psoriatic Arthritis"[ti] OR "Psoriasis Arthropathica"[ti] OR "Psoriatic Arthropathy"[ti] OR "Psoriatic Arthropathies"[ti] OR **"Arthritis, Juvenile"[majr]** OR "juvenile idiopathic arthritis"[ti] OR "Juvenile Arthritis"[ti] OR "Juvenile Chronic Arthritis"[ti] OR "Juvenile Enthesitis-Related Arthritis"[ti] OR "Juvenile Idiopathic Arthritis"[ti] OR "Juvenile Oligoarthritis"[ti] OR "Juvenile Psoriatic Arthritis"[ti] OR "Juvenile Rheumatoid Arthritis"[ti] OR "Juvenile Systemic Arthritis"[ti] OR "Juvenile-Onset Still's Disease"[ti]) AND ("Fitness Trackers"[Mesh] OR "Fitness Trackers"[tw] OR "Fitness Tracker"[tw] OR "Fitness Tracking"[tw] OR "wearable activity tracker"[tw] OR "wearable activity trackers"[tw] OR "wearable activity tracking"[tw] OR "activity tracker"[tw] OR "activity trackers"[tw] OR "activity tracking"[tw] OR "pedometer"[tw] OR "pedometers"[tw] OR pedomet*[tw] OR "Wearable Electronic Devices"[Mesh:NoExp] OR "wearables"[tw] OR "wearable"[tw] OR wearab*[tw] OR "electronic skin"[tw] OR "Monitoring, Ambulatory"[Mesh:NoExp] OR "Ambulatory Monitoring"[tw] OR "Fitbit Flex"[tw] OR "Smartwatch"[tw] OR "Smartwatches"[tw] OR "Smart watch"[tw] OR "Smart watches"[tw] OR "Actigraphy"[Mesh] OR "Actigraphy"[tw] OR Actigra*[tw] OR "tracker"[tw] OR "trackers"[tw] OR "step counter"[tw] OR "step counters"[tw] OR stepcount*[tw] OR "step count*"[tw] OR "Digi-Walker"[tw] OR "Walk4Life"[tw] OR "TrekLINQ"[tw] OR "App"[tw] OR "Apps"[tw] OR "FitBit"[tw] OR "Garmin"[tw] OR "Misfit"[tw] OR "Omron"[tw] OR "Apple Watch"[tw] OR "Apple Watches"[tw] OR "Mobile Applications"[Mesh] OR "Mobile Applications"[tw] OR "Mobile Application"[tw] OR "Portable Electronic Application"[tw] OR "Portable Electronic Applications"[tw] OR "Portable Software Application"[tw] OR "Portable Software Applications"[tw] OR (("Accelerometry"[mesh] OR accelero*[tw] OR monitor*[tw]) AND ("Cell Phone"[Mesh:NoExp] OR "cell phone"[tw] OR "cell phone"[tw] OR "smartphone"[tw] OR "smartphones"[tw] OR "smart phone"[tw] OR "smart phones"[tw] OR "telephone"[tw] OR "telephones"[tw] OR "mobile phone"[tw] OR "mobile phones"[tw] OR "gps"[tw] OR "global positioning systems"[tw] OR "global positioning system"[tw]))) AND ("2000/01/01"[PDAT] : "3000/12/31"[PDAT]))**)**
