# Supplementary material for: Reporting quality of interventions using a wearable activity tracker to improve physical activity in patients with inflammatory arthritis or osteoarthritis: a systematic review
Source: Rheumatol Int. 2022 Dec 1;43(5):803–24. doi: 10.1007/s00296-022-05241-x (PMC10073167; doi:10.1007/s00296-022-05241-x)
Supplement: Supplementary file 2 — Supplementary file2 (DOCX 18 KB) [file 296_2022_5241_MOESM2_ESM.docx]

Article title: Reporting quality of interventions using a wearable activity tracker to improve physical activity in patients with inflammatory arthritis or osteoarthritis: a systematic review

Journal: Rheumatology International

M.A.T. van Wissen^1^*, M.A.M. Berger^2^, J.W. Schoones^3^, M.G.J. Gademan^1, 4^, C.H.M. van den Ende^5,6^, T.P.M. Vliet Vlieland^1^, S.F.E. van Weely^1^

1.Department of Orthopaedics, Rehabilitation and Physical Therapy, Leiden University Medical Center, Leiden, The Netherlands; 2.The Hague University of applied sciences, The Hague, The Netherlands; 3. Directorate of Research Policy (Walaeus Library), Leiden, The Netherlands;4. Department of Clinical Epidemiology, Leiden University Medical Center, Leiden, The Netherlands; 5. Department of Research, Sint Maartenskliniek, Nijmegen, The Netherlands; 6.Department of Rheumatology, Radboud University Medical Center, Nijmegen, The Netherlands

*Corresponding author: M.A.T. van Wissen. m.a.t.van_wissen@lumc.nl

**Supplementary Table S2: Overlap between CERT and CONSORT E-Health checklist items**

| **CERT item** | **Description** | **CONSORT E-Health item** | **Description** |
| --- | --- | --- | --- |
| 1 | Detailed description of the type of exercise equipment (e.g., weights, exercise equipment such as machines, treadmill, bicycle ergometer, etc.). | I | I: Mention names, credential, affiliations of the developers, sponsors, and owners of the software |
| 2 | Detailed description of the qualifications, teaching/supervising expertise and/or training undertaken by the exercise instructor. | X | X: Clarify the level of human involvement: Detail number and expertise of professionals involved |
| 3 | Describe whether exercises are performed individually or in a group. | VII | VII: Access: Whether they had to be a member of specific group |
| 4 | Describe whether exercises are supervised or unsupervised and how they are delivered. | X XI | X: Clarify the level of human involvement: as well as “type of assistance offered, the timing and frequency of the support, how it is initiated, and the medium by which the assistance is delivered” XI: Report any prompts/reminders used: Clarify if there were prompts (letters, emails, phone calls, SMS) to use the application, what triggered them, frequency, etc. |
| 5 | Detailed description of how adherence to exercise is measured and reported. | 0 |  |
| 6 | Detailed description of motivation strategies. | VIII XI XII | VIII: Describe mode of delivery:  XI: Report any prompts/reminders used: Clarify if there were prompts (letters, emails, phone calls, SMS) to use the application, what triggered them, frequency, etc.  XII: Describe any co-interventions (incl. training/support): Clearly state any interventions that are provided in addition to the targeted eHealth intervention, as Ehealth intervention may not be designed as standalone intervention |
| 7a | Detailed description of the decision rule(s) for determining exercise progression. | VIII | VII: Describe mode of delivery: and the theoretical framework used to design the intervention and in-depth description of the content (including where it is coming from and who developed it) |
| 7b | Detailed description of how the exercise program was progressed (e.g., numbers of repetitions, resistance, load, speed, etc.). | IX | IX: Describe use parameters: Clarify what instructions or recommendations were given to the user, e.g., regarding timing, frequency, heaviness of use, if any, or was the intervention used ad libitum. |
| 8 | Detailed description of each exercise to enable replication (e.g., photographs, illustrations, video, Smartphone app, website, protocol paper, etc.). | V | V: Ensure replicability: by publishing the source code, and/or providing screenshots/screen-capture video |
| 9 | Detailed description of any home program component  (e.g., other exercises, stretching, functional tasks, etc.). | XII | XII: Describe any co-interventions (incl. training/support): Clearly state any “interventions that are provided in addition to the targeted eHealth intervention” |
| 10 | Describe whether there are any non-exercise components (e.g., training or information materials, education, cognitive–behavioral therapy, massage, etc.). | VIII XII | VIII: Describe mode of delivery: and the theoretical framework used to design the intervention (instructional strategy, behavior change techniques, persuasive features, etc.) XII: Describe any co-interventions (incl. training/support): Clearly state any “interventions that are provided in addition to the targeted eHealth intervention” |
| 11 | Describe the type and number of adverse events that occur during exercise. | 0 |  |
| 12 | Describe the setting in which the exercises are performed. | VII | VII: Access: Describe how participants accessed the application, in what setting/context. |
| 13 | Detailed description of the exercise intervention including, but not limited to, number of exercise repetitions/sets/sessions, session duration, program duration, etc. | IX | IX: Describe use parameters: Clarify what instructions or recommendations were given to the user, e.g., regarding timing, frequency, heaviness of use |
| 14a | Describe whether the exercises are generic (one size fits all) or tailored. | VIII | VIII: Describe mode of delivery: whether [and how] it is tailored to individual circumstances and allows users to track their progress and receive feedback |
| 14b | Detailed description of how exercises are tailored to the individual. | VIII IX | VIII: Describe mode of delivery: whether [and how] it is tailored to individual circumstances and allows users to track their progress and receive feedback IX: Describe use parameters: Clarify what instructions or recommendations were given to the user, e.g., regarding timing, frequency, heaviness of use |
| 15 | Describe the decision rule for determining the starting level at which people start an exercise program (e.g., beginner, intermediate, advanced, etc.). | 0 |  |
| 16a | Describe how adherence or fidelity to the exercise intervention is assessed/measured. | 0 |  |
| 16b | Describe the extent to which the intervention was delivered as planned. | 0 |  |
