# Supplementary material for: Reporting quality of interventions using a wearable activity tracker to improve physical activity in patients with inflammatory arthritis or osteoarthritis: a systematic review
Source: Rheumatol Int. 2022 Dec 1;43(5):803–24. doi: 10.1007/s00296-022-05241-x (PMC10073167; doi:10.1007/s00296-022-05241-x)
Supplement: Supplementary file 3 — Supplementary file3 (DOCX 42 KB) [file 296_2022_5241_MOESM3_ESM.docx]

Article title: Reporting quality of interventions using a wearable activity tracker to improve physical activity in patients with inflammatory arthritis or osteoarthritis: a systematic review

Journal: Rheumatology International

M.A.T. van Wissen^1^*, M.A.M. Berger^2^, J.W. Schoones^3^, M.G.J. Gademan^1, 4^, C.H.M. van den Ende^5,6^, T.P.M. Vliet Vlieland^1^, S.F.E. van Weely^1^

1.Department of Orthopaedics, Rehabilitation and Physical Therapy, Leiden University Medical Center, Leiden, The Netherlands; 2.The Hague University of applied sciences, The Hague, The Netherlands; 3. Directorate of Research Policy (Walaeus Library), Leiden, The Netherlands;4. Department of Clinical Epidemiology, Leiden University Medical Center, Leiden, The Netherlands; 5. Department of Research, Sint Maartenskliniek, Nijmegen, The Netherlands; 6.Department of Rheumatology, Radboud University Medical Center, Nijmegen, The Netherlands

*Corresponding author: M.A.T. van Wissen. m.a.t.van_wissen@lumc.nl

**Supplementary Table S3 Description of WAT and PA program of included studies** **in a systematic review on interventions promoting PA in patients with inflammatory arthritis or osteoarthritis**

|  | **Device** |  |  | **PA program explanation** |  |  |  |  |
| --- | --- | --- | --- | --- | --- | --- | --- | --- |
|  | **Brand/Type WAT** | **Location of wear** | **Daily wearing time and duration (T)** | **Determining starting level PA program** | **Description PA program** | **Tailoring of PA program  (individual or generic)** | **Progression rule of PA program** | **Description of progression PA program** |
| **CERT item** | **1** | **-** | **-** | **15** | **13** | **14a/14b** | **7a** | **7b** |
| **CONSORT E-HEALTH item** | **5 I** | **-** | **-** | **-** | **5 IX** | **5 VIII 5 IX** | **5 VIII** | **5 IX** |
| **Labat, 2022, France [52]** | Garmin Vívofit 4.0 | Wrist | Not described T1 =1-12 weeks  T2 = 24-36 wees | Not described | The participants were asked to schedule 2 weekly sessions of PA of their choice, with Nordic walking given encouragement. The patients were monitored by a WAT, for the tracking of the number of steps, distance covered, and calories burned. | Individual: Each individual participants were also able to set a personalized daily step goal. | Not described | Not described |
| **Plumb Vilardage, 2022, United States [44]** | Garmin VivoFit 4.0 | Wrist | Upon waking and until bedtime daily T = 6 weeks | Not described | Participants first discussed the fitness tracker and  their baseline level of daily steps. | Individual: participants first discussed the fitness tracker and their baseline level of daily steps. The study therapist then explored the participant’s experience with OA pain and physical activity and introduced personal values and activity-rest cycle skills. | Not described | Not described |
| **Ostlind, 2021, Sweden [43]** | Fitbit Flex 2, Inc, San Francisco, CA, USA | Wrist | From morning until bedtime T = 12 weeks | Not described | Each participant met with physical therapist and received the Fitbit. They were aided in installing the Fitbit application (app). The default activity goal for the Fitbit of 10,000 steps per day was changed to 7,000 in order to make it more achievable for this population with hip and/or knee OA. Previous research has also suggested that 7,000 steps per daymight be an accurate estimate for meeting the recommended 150 min per week of MVPA. Therewere also other default activity goals in the app; distance (8.05 km), calories burned (based on gender and weight) and bouted active minutes (30 min). Participants were asked not to change them. | Generic: The default activity goal for the Fitbit of 10,000 steps per daywas changed to 7,000 in order to make it more achievable for this population with hip and/or knee OA. Previous research has also suggested that 7,000 steps per day might be an accurate estimate for meeting the recommended 150 min per week of MVPA  Individual: Therewere also other default activity goals in the app; distance (8.05 km), calories burned (based on gender and weight) and bouted active minutes (30 min). Participants were asked not to change them. | Not described | Not described |
| **Christiansen, 2020, United States [48]** | Fitbit Zip, Fitbit, Inc,  San Francisco, CA, USA | Waist at the right anterior  superior iliac crest | Daily (during walking hours) T = 6 months | Not described | The end goal was to walk at least 6,000 steps/day until discharge. If the participant achieved 6,000 steps/day, they were encouraged to continue to increase their steps/day since health benefits persist with more PA. | Individual: The participant had a personal PA goal set with a physical therapist. | Several factors were considered to progress the steps/day goal, including if the weekly steps/day goal was achieved in at least 4 of the last 7 days, the participant’s current health status, the physical therapist’s clinical judgment, and the participant’s personal PA goal. | A research assistant contacted participants in the intervention group once a month for 6 months and continued jointly setting steps/day goals with the study participant after discharge from PT. |
| **Li, 2020a, Canada [38]** | Fitbit Flex 2, Inc, San Francisco, CA, USA FitViz,Inc, San Francisco, CA, USA | Wrist | Not described T = 8 weeks | Not described | physical therapist set the parameters on their assigned participants’ FitViz accounts based on the participant’s goals. These parameters included the following: (1) the upper and lower limits of intensity and duration of MVPA (i.e., to promote physical activity based on the participant’s goal), (2) the duration when a sedentary behavior should be interrupted (i.e., to promote less sitting), and (3) the rest time in between sessions of MVPA (i.e., to promote pacing). | Individual: During the individual counseling, physical therapist used the Brief Action Planning approach to guide participants to set SMART physical activity goals. Individual parameters included the following: (1) the upper and lower limits of intensity and duration of MVPA (i.e., to promote physical activity based on the participant’s goal), (2) the duration when a sedentary behavior should be interrupted (i.e., to promote less sitting), and (3) the rest time in between sessions of MVPA (i.e., to promote pacing). | Not described | Not described |
| **Li, 2020b, Canada [53]** | Fitbit Flex 2, Inc, San Francisco, CA, USA  FitViz,Inc, San Francisco, CA, USA | Not described | Not described T = 8 weeks | Not described | Based on the participant’s goal, the physical therapist then set the physical activity parameters on FitViz to match the individual’s activity plan, thereby providing automated personalized feedback on goal attainment. These parameters included: 1) the upper and lower bound of intensity and duration of MVPA (i.e., to promote physical activity based on the individual’s goal), 2) the upper limit of time in continuous sedentary behavior (i.e., to promote less sitting), and 3) the rest time between sessions of MVPA (i.e., to promote pacing). | Individual: Based on the participant’s goal, the physical therapist then set the physical activity parameters on FitViz to match the individual’s activity plan, thereby providing automated personalized feedback on goal attainment. These parameters included: 1) the upper and lower bound of intensity and duration of MVPA (i.e., to promote physical activity based on the individual’s goal), 2) the upper limit of time in continuous sedentary behavior (i.e., to promote less sitting), and 3) the rest time between sessions of MVPA (i.e., to promote pacing). | Not described | Not described |
| **Zaslavsky, 2019, United States [47]** | Fitbit Charge 2, Inc, San Francisco, CA, USA | Wrist | Not described T = 14 weeks | Over the first week, average step count was calculated and constituted participants’ baseline. | The step count attainment was determined at 3 time points and calculated as follows. Over the first week, average step count was calculated and constituted participants’ baseline. Over the next month, departure from that baseline was calculated each week and categorized according to the extent of percentage difference between baseline and that week average step count. Categories included “decline of more than 5% from baseline”, “maintenance within 5%,” and “increase of more than 5% from baseline”. Weekly categories (i.e., ‘decline’, ‘maintenance’, ‘increase’) informed content of the weekly messages and provided reinforcement for those who increased or maintained, and encouragement for those in a ‘decline’ category. After one month, a new baseline count was calculated according to the average step count over the first month. Accordingly, subsequent weekly text messages over the second month reacted to the percentage differences between weekly step counts and a recalibrated one-month baseline. Over the third month, this procedure was repeated again. | Individual: Personalized intervention informed by control theory framework. | Not described | The step count attainment was determined at 3 time points and calculated as follows. Over the first week, average step count was calculated and constituted participants’ baseline. Accordingly, subsequent weekly text messages over the second month reacted to the percentage differences between weekly step counts and a recalibrated one-month baseline. Over the third month, this procedure was repeated again. Upon completion of the intervention, a total of 12 motivational text messages were sent to the participants. |
| **Li, 2018, Canada [40]** | Fitbit Flex, Inc, San Francisco, CA, USA | Wrist of the nondominant side | 24 hours a day except during water-based activity or when charging T = 13 weeks | Not described | The physical activity data were wirelessly synchronized with Fitbit’s online Dashboard that could be viewed only by the participants and their study physical therapist. During the intervention period, the physical therapist reviewed the participant’s physical activity on the Dashboard and progressively modified their SMART goals during four biweekly 20-minute phone calls. | Individual: The individual counseling portion followed the Brief Action Planning approach, whereby physical therapist guided participants to identify activity goals,develop an action plan, and identify barriers and solutions. The physical therapist used the SMART (specific, measurable, attainable, relevant,time-bound) principle during goal setting (e.g., 30 minutes ofbrisk walking in the neighborhood in the evening three times aweek). Participants were then asked to rate their confidence inexecuting the plan on a zero to 10 scale, with 10 meaning very confident. The process was repeated until the confidence rating reached 7 or higher out of 10. For sedentary behaviors, the physical therapist began by asking participants to estimate their sitting time in anormal day and identify ways to break up the sitting time. They then repeated the goal setting and confidence assessment. | Not described | The physical activity data were wirelessly synchronized with Fitbit’s online Dashboard that could be viewed only by the participants andtheir study physical therapist. During the intervention period, the physical therapist reviewed the participant’s physical activity on the Dashboard and progressively modified their SMART goals during four biweekly 20-minute phone calls. |
| **Paxton, 2018, United States [50]** | Fitbit Zip, Inc, San Francisco, CA, USA | Not described | Not described T = 12 weeks | Not described | Each week, a researcher called each participant to address and alleviate any participant- reported barriers to physical activity goal achievement. Physical activity goals were modified weekly, as necessary, and the goal of the research staff was to negotiate a physical activity goal of 5% greater than the previous week when compared to the mean physical activity performed during the previous week. The goal of 5% increase was used as it is defined as a ‘moderate’ increase in physical activity. | Individual: Participants randomized to the physical activity feedback program were prescribed a daily physical activity goal and oriented to the use of the Fitbit real-time physical activity wearable sensor (Fitbit Zip, San Francisco, CA) by which participants were able to self-assess their daily level of physical activity with visual feedback. | The goal of 5% increase was used as it is defined as a ‘moderate’ increase in physical activity. | Physical activity goals were modified weekly, as necessary, and the goal of the research staff was to negotiate a physical activity goal of 5% greater than the previous week when compared to the mean physical activity performed during the previous week. |
| **Darabseh, 2017, Jordan [49]** | Omron HJ-320; Omron Healthcare, Inc., Bannockburn, IL, USA | Not described | Not described T = 7 days | Not described | Not described | Not described | Not described | Not described |
| **Katz, 2017, United States [51]** | Fitbit Zip, Fitbit, Inc, San Francisco, CA, USA | Not described | Time they got out of bed in the morning until they went to bed at night, except while showering, bathing, or swimming. T = 21 weeks | Step targets were based on the week of activity  monitoring between the baseline and randomization visits. | At each 2-week follow up, the intervention group’s average daily steps were compared with the target assigned for that period. If individuals met their target, a new step target was calculated to increase steps by 10% from the previous target. If individuals did not meet the target, the existing target remained in place. | Individual: At each 2-week follow up, the intervention group’s average daily steps were compared with the target assigned for that period. If individuals met their target, a new step target was calculated to increase steps by 10% from the previous target. If individuals did not meet the target, the existing target remained in place. | Not described | At each 2-week follow up, the intervention group’s average daily steps were compared withthe target assigned for that period. If individuals met their target, a new step target was calculated to increase steps by 10% from the previous target. If individuals did not meet the target, the existing target remained in place. |
| **Li, 2017, Canada [39]** | Fitbit Flex, Inc, San Francisco, CA, USA | Wrist of the nondominant side | To wear the fitness band 24 hours a day except during water-based activity or when charging the device.  T = 2 months | Not described | The data were wirelessly synchronized with Fitbit’s online dashboard that could be viewed only by the participants and their study physical therapist. During the intervention period, the physical therapist reviewed each individual’s physical activity on the dashboard and progressively modified the activity goals during 4 weekly 20-minute telephone calls. | Individual: The counselling component followed the brief action planning approach, whereby the physical therapist guided participants to identify their activity goals, develop an action plan, identify barriers and solutions, and then rate their confidence in executing the plan. The process was repeated until the confidence rating reached at least 7 out of 10, indicating that the person was confident about implementing the plan. For sedentary behavior, the physical therapist began by asking participants to estimate their sitting time in a normal day and identify ways to break up the sitting time. They then repeated the goal setting and confidence assessment. | Not described | Not described |
| **Skrepnik, 2017, United States [45]** | Jawbune UP 24 OA GO app | Wrist | Remove the monitor only during the weekly charging times and in situations where the device would be submerged in water.  T = 90 days | Daily step goal based on patient’s baseline steps per day during screening. | The OA GO app (downloaded to a trial-sponsored iPhone 5 or newer) provided motivational messages and requested that the patient enter pain and mood data on a once-daily basis. Trial coordinators demonstrated app use, provided charging instructions for the Jawbone UP 24, and set the daily step goal. | Individual: Set the daily step goal based on patient’s baseline steps per day during screening. | Not described | Not described |
| **Hiyama, 2011, Japan [37]** | Pedometer, KenzLifecoder EX, Suzuken Co.,Ltd.,  Nagoya, Aichi, Japan. | Waist belt | At all times except when at home T = 4 weeks | Before baseline assessments, the number of steps walked daily was recorded with a pedometer for a week. The walking group was instructed to increase their daily steps to 3000 more than their number of steps (median) before baseline assessments. | The walking group was instructed to increase their daily steps to 3000 more than their number of steps (median) before baseline assessments. | Individual: The walking group was instructed to increase their daily steps to 3000 more than their number of steps (median) before baseline assessments. | Not described | To increase their daily steps to 3000 more than their number of steps (median) before baseline assessments. |
| **Ng, 2010, Australia [41]** | Pedometer, no brand | Not described | Not described  T = 12 weeks | Not described | Participants were asked to initially walk at least 1500 steps (approximately 15 minutes) on each ‘walking’ day in addition to any walking they were currently doing, and to do this additional walking in a single session. They were asked to increase from 1500 steps to 3000 steps (approximately 30 minutes) by the Week 12 assessment and, to accommodate participants who were unable to walk this amount continuously,were advised that the walks could be done in bouts of at least 1500 steps each. At the Week 12 session, participants were asked to increase their walking to 6000 steps (approximately 60 minutes) by Week 18, the end of the intervention. | Generic | Not described | Participants were asked to initially walk at least 1500 steps (approximately 15 minutes) on each ‘walking’ day in addition to any walking they were currently doing, and to do this additional walking in a single session. They were asked to increase from 1500 steps to 3000 steps (approximately 30 minutes) by the Week 12 assessment and, to accommodate participants who were unable to walk this amount continuously,were advised that the walks could be done in bouts of at least 1500 steps each. At the Week 12 session, participants were asked to increase their walking to 6000 steps (approximately 60 minutes) by Week 18, the end of the intervention. |
| **Talbot, 2003, United States [46]** | Pedometer, no brand | Not described | Not described T = 12 weeks | The number of daily steps was modified to the individuals’ baseline step count | The number of daily steps was modified to the individuals’ baseline step count and increased by 10% every 4 weeks. | Individual: The number of daily steps was modified to the individuals’ baseline step count and increased by 10% every 4 weeks. | Not described | The number of daily steps was modified to the individuals’ baseline step count and increased by 10% every 4 weeks. |

*Abbreviations and explanatory:
WAT= Wearable Activity Tracker, PA=Physical Activity, PT=Physical Therapy, T=duration, MVPA=Moderate to Vigorous Physical Activity, OA=Osteoarthritis, SMART=Specific, Measurable, Achievable, Realistic and Timely.*
