# Supplementary material for: Reporting quality of interventions using a wearable activity tracker to improve physical activity in patients with inflammatory arthritis or osteoarthritis: a systematic review
Source: Rheumatol Int. 2022 Dec 1;43(5):803–24. doi: 10.1007/s00296-022-05241-x (PMC10073167; doi:10.1007/s00296-022-05241-x)
Supplement: Supplementary file 4 — Supplementary file4 (DOCX 45 KB) [file 296_2022_5241_MOESM4_ESM.docx]

Article title: Reporting quality of interventions using a wearable activity tracker to improve physical activity in patients with inflammatory arthritis or osteoarthritis: a systematic review

Journal: Rheumatology International

M.A.T. van Wissen^1^*, M.A.M. Berger^2^, J.W. Schoones^3^, M.G.J. Gademan^1, 4^, C.H.M. van den Ende^5,6^, T.P.M. Vliet Vlieland^1^, S.F.E. van Weely^1^

1.Department of Orthopaedics, Rehabilitation and Physical Therapy, Leiden University Medical Center, Leiden, The Netherlands; 2.The Hague University of applied sciences, The Hague, The Netherlands; 3. Directorate of Research Policy (Walaeus Library), Leiden, The Netherlands;4. Department of Clinical Epidemiology, Leiden University Medical Center, Leiden, The Netherlands; 5. Department of Research, Sint Maartenskliniek, Nijmegen, The Netherlands; 6.Department of Rheumatology, Radboud University Medical Center, Nijmegen, The Netherlands

*Corresponding author: M.A.T. van Wissen. m.a.t.van_wissen@lumc.nl

**Supplementary Table S4 Type and characteristic of non exercise components or motivational strategies in PA programs of included studies in a systematic review on interventions promoting PA in patients with inflammatory arthritis or osteoarthritis**

|  | **Type of non exercise component/ motivational strategy** | **Individual/ group setting of  non exercise component** | **Supervised/unsupervised of  non exercise component** | **Number of sessions of  non exercise component** | **Setting of  non exercise component** | **Description of the person who delivered the non  exercise component or motivational strategy** | **Training of the person who delivered the non  exercise component or motivational strategy** |
| --- | --- | --- | --- | --- | --- | --- | --- |
| **CERT item** | **10, 6** | **3** | **4** | **13** | **9,12** | **2** | **2** |
| **CONSORT E-health item** | **5 VIII 5 XI 5 XII** | **5 VII** | **5 X 5 XI** | **5 IX** | **5 VII 5 XII** | **5 X** | **5 X** |
| **Labat, 2022, France [52]** | a) Instruction/training on how to perform PA of their choice (C and E group)  b) Activity reminders (C group) c) Coach-supervised PA (C and E group) | a) Not described b) Individual c) Not described | a) Supervised (face-to-face) b) Unsupervised (SMS) c) Supervised (face-to-face) | a) Not described b) 12 weeks, weekly  c) 12 weeks, 1 hour per week | a) Not described b) Not described c) Not described | a) Presence of physiotherapists and doctors knowledgeable on the rehabilitative management of patients b) Not described c) Not described | Not described |
| **Plumb Vilardage, 2022, United States [44]** | a) Study workbook (E group) and 2 treatment session to identify personal values related to PA and another life domain (E group)  c) Handout on the use of the Garmin device and tips how to safely engage in PA outdoors (C group) | a) Individual  b) Individual | a) Supervised ( phone call) b) Unsupervised (written instruction) | a) 2 session, 45 minutes  b) Not described | a) Not described b) Not described | a) A master’s level study therapist b) Not described | A master’s level study therapist guided by a written treatment manual, audio recorded, and checked for fidelity by a senior researcher. |
| **Ostlind, 2021, Sweden [43]** | Supported Osteoarthritis Self-management Program (SOASP): a) Information about OA, exercises and self-management  b) Individual appointment with a PT and introduced to specific exercise based on their needs and goals  c) Supervised group training d) Aiding in stalling the Fitbit app and synchronizing the device to the participants app | a) Group b) Individual  c) Group d) Individual | a) Supervised (face-to-face) b) Supervised (face-to-face) c) Supervised (face-to-face) d) Supervised (face-to-face) | a) Two times b) One time  c) Two times a week for six weeks  d) Not described | a) Not described b) Not described c) Healthcare centers or physiotherapy clinics D) Not described | a) Physical therapist b) Physical therapist c) Occupational therapists or dieticians  d) Physical therapist | Not described |
| **Christiansen, 2020, United States [48]** | a) Instructions on how to set up, use, and sync the Fitbit Zip to their smartphone, tablet, or home computer using the app provided by Fitbit. b) PA goal setting | a) Individual b) Individual | a) Supervised (face-to-face instruction) and unsupervised (written instruction) b) Supervised (phone call) | a) Not described b) 1 per month, 6 months | a) Not described  b) Home | a) A licensed physical therapist b) Research assistant | Not described |
| **Li, 2020a, Canada [38]** | a) Group education focused on physical activity in OA management and strategies to manage joint symptoms b) Personal counseling (Brief Action Planning approach and setting parameters on FitViz app) c) Physical therapist counseling PA goals | a) Group b) Individual c) Individual | a) Supervised (face-to-face) b) Supervised (face-to-face) c) Supervised (phone call) | a) Not described,20 minutes b) Not described, 30 minutes c) Biweekly, 8 weeks, 20-30 minutes | a) Meeting room at either the Mary Park Arthritis Centre, Fraser Health Authority, or Arthritis Research Canada b) Meeting room at either the Mary Park Arthritis Centre, Fraser Health Authority, or Arthritis Research Canada c) Home | a) A trained physical therapist  b) A trained physical therapist c) A trained physical therapist | Physical therapists completed a 2-day basic training in motivational interviewing at the University of British Columbia. In addition, they attended an orientation session, received a counseling guide, and shadowed at least one education and counseling session before they were paired with a participant. |
| **Li, 2020b, Canada [53]** | a) Education  b) Personal counseling (Brief Action Planning approach and setting parameters on FitViz app) c) Physical therapist counseling PA goals | a) Group b) Individual c) Individual | a) Supervised (face-to-face) b) Supervised (face-to-face) c) Supervised (phone call) | a) Not described,20 minutes b) Not described, 30 minutes c) Biweekly, 8 weeks, 20-30 minutes | a) Meeting room at either the Mary Park Arthritis Centre, Fraser Health Authority, or Arthritis Research Canada b) Meeting room at either the Mary Park Arthritis Centre, Fraser Health Authority, or Arthritis Research Canada c) Home | a) A trained physical therapist  b) A trained physical therapist c) A trained physical therapist | 2-day training in motivational interviewing offered by the University of British Columbia. In addition, they attended an orientation session, received a counseling guide, and shadowed at least 1 education and counseling session before they were paired with a participant. During the training and orientation, physical therapists practiced using the brief action planning approach to set (SMART) goals with their peers or a model participant. |
| **Zaslavsky, 2019, United States [47]** | a) Motivational text messages (provided motivational feedback according to their adaptive step count attainment) b) Motivational interviewing (focused on specific goal setting and action planning behavioral change techniques derived from the control theory framework) c) A basic orientation when enrolled into the study, assistance with troubleshooting for the next 14 weeks, and limited assistance after that. | a) Individual  b) Individual c) Individual | a) Supervised (by text message) b) Supervised (by phone-call) c) supervised (by phone-call) | a) Weekly (total of 12 messages) b) Three times (week 1, 5 and 9) c) 14 days | a) Home b) Home c) Home | a) The iCardia research platform  b) Interventionist c) Interventionist | An interventionist, who delivered phone calls, received training in Motivational Interviewing. |
| **Li, 2018, Canada [40]** | a) Standard education about PA b) Counseling in PA c) Reviewing participants PA SMART goals | a) Group (2-4 participants) b) Individual c) Individual | a) Supervised (face to face) b) Supervised (face to face) c) Phone call | a) 1 session (15 minutes) b) 1 session c) 4 biweekly (20 minutes) | a) Not described  b) Not described c) Home | a) Physical therapist  b) Physical therapist c) Physical therapist | The physical therapists attended a 2-day introductory motivational interview course offered by the University of British Columbia Extended Learning program. Before data collection, we held two orientation sessions (2 hours each) for the physical therapists to review the study protocol and practice the counseling component. |
| **Paxton, 2018, United States [50]** | a) Newsletter b) Physical activity guidelines c) Notes regarding future meetings d) Address and alleviate any participant-reported barriers to PA goal achievement e) Physical activity monitor with instruction on use | a) Group b) Group  c) Group d) Individual e) Individual | a) Not described b) Not described  c) Not described d) Supervised (phone call) e) Supervised (face to face) | a) Monthly b) Monthly c) Monthly  d) Individual e) Once during first assessment | a) A meeting space central to participants’ locations b) A meeting space central to participants’ locations c) A meeting space central to participants’ locations d) Home e) Clinical research laboratory | a) Research staff b) Research staff c) Research staff d) Research staff  e) Research staff | Not described |
| **Darabseh, 2017, Jordan [49]** | Usual physiotherapy program for the first 7 days after the surgery | Not described | Not described | Not described | Not described | Not described | Not described |
| **Katz, 2017, United States [51]** | a) An educational brochure (Be Active Your Way: A Guide for Adults [http://health.gov/paguidelines/pdf/ paguide.pdf])  b) A guided discussion of simple ways to increase physical activity in daily life based on the booklet  c) Step diary, and individualized daily step target feedback | a) Group b) Group c) Individual | a) Supervised  b) Supervised  c) Supervised (phone call) | a) Not described  b) Not described c) Every two week | a) Not described  b) Not described c) Home | a) Not described b) Not described c) Not described | Not described |
| **Li, 2017, Canada [39]** | a) Standardized group education session about PA  b) PA goal setting  c) Activity counselling | a) Group (2-3 participants) b) Not described  c) Individual | a) Supervised  b) Supervised c) Supervised (phone-call) | a) Not described, in total 1.5 hours with the PA goal setting b) Not described, in total 1.5 hours with the group education c) 4 weekly (2 minutes) | a) Not described b) Not described c) Home | a) Physical therapist b) Physical therapist c) Physical therapist | Not described |
| **Skrepnik, 2017, United States [45]** | a) Hylan G-F 20  b) Information on the benefits of walking in a brochure available from the Arthritis Foundation) c) The OA GO app (downloaded to a trial-sponsored iPhone 5 or newer) provided motivational messages and requested that the patient enter pain and mood data on a once-daily basis. d) App use, provided charging instructions for the Jawbone UP 24, and set the daily step goal based | a) Not described b) Not described c) Not described d) Not described | a) Not described b) Not described c) Not described d) Supervised | a) Not described b) Not described c) Not described d) Not described | a) Not described b) Not described c) Not described d) Not described | a) Not described b) Not described c) Not described d) Trial coordinator | Not described |
| **Hiyama, 2011, Japan [37]** | Physical therapy (ice therapy, range of motion exercises and muscle strengthening exercises | Individual | Supervised | Once a week | Home | Not described | Not described |
| **Ng, 2010, Australia [41]** | a) A walking guide b) Log sheet for recording daily step counts c) Glucosamine Sulphate intake d) Planner for scheduling walking sessions | a) Individual b) Individual c) Not described d) Individual | a) Supervised b) Supervised c) Not described d) Supervised | a) Not described b) Weekly c) Weekly d) Weekly | a) Not described  b) Not described  c) Not described  d) Not described | a) Interventionist b) Interventionist c) Interventionist d) Interventionist | A doctoral student with training in exercise science and physical activity behavior change. |
| **Talbot, 2003, United States [46]** | a) Arthritis self-management program including a 1-hour unit on exercise as a component of arthritis self-management b) Brief individual counseling the pedometer logs were reviewed and feedback provided. c) A booklet explaining the principles of exercise, including warm-up, cool-down, stretching, and such arthritis principles as the 2-hour pain rule and balancing rest with activity | a) Not described b) Individual  c) Not described | a) Supervised b) Supervised  c) Not described | a) 12-hours, not described b) < 5 minutes, not described c) Not described | a) Not described b) Not described c) Not described | a) Registered nurses  b) Registered nurses  c) Registered nurses | Registered nurses attended the Arthritis Foundation’s 16-hour training course and conducted all classes. |

*Abbreviations and explanatory:
PA=Physical Activity, PT=Physical Therapy, OA=Osteoarthritis, SMART=* Specific, Measurable, Attainable, Relevant, and time-bound, C group= control group, E group= intervention group
