# Supplementary material for: Reporting quality of interventions using a wearable activity tracker to improve physical activity in patients with inflammatory arthritis or osteoarthritis: a systematic review
Source: Rheumatol Int. 2022 Dec 1;43(5):803–24. doi: 10.1007/s00296-022-05241-x (PMC10073167; doi:10.1007/s00296-022-05241-x)
Supplement: Supplementary file 5 — Supplementary file5 (DOCX 36 KB) [file 296_2022_5241_MOESM5_ESM.docx]

Article title: Reporting quality of interventions using a wearable activity tracker to improve physical activity in patients with inflammatory arthritis or osteoarthritis: a systematic review

Journal: Rheumatology International

M.A.T. van Wissen^1^*, M.A.M. Berger^2^, J.W. Schoones^3^, M.G.J. Gademan^1, 4^, C.H.M. van den Ende^5,6^, T.P.M. Vliet Vlieland^1^, S.F.E. van Weely^1^

1.Department of Orthopaedics, Rehabilitation and Physical Therapy, Leiden University Medical Center, Leiden, The Netherlands; 2.The Hague University of applied sciences, The Hague, The Netherlands; 3. Directorate of Research Policy (Walaeus Library), Leiden, The Netherlands;4. Department of Clinical Epidemiology, Leiden University Medical Center, Leiden, The Netherlands; 5. Department of Research, Sint Maartenskliniek, Nijmegen, The Netherlands; 6.Department of Rheumatology, Radboud University Medical Center, Nijmegen, The Netherlands

*Corresponding author: M.A.T. van Wissen. m.a.t.van_wissen@lumc.nl

**Supplementary Table S5 Adherence, fidelity and adverse events in included studies in a systematic review on interventions promoting PA in patients with inflammatory arthritis or osteoarthritis**

|  | **How adherence of the participant to exercise intervention is measured** | **The extent to which the intervention was completed by the participant** | **How fidelity of the instructor is assessed after the basic training** | **The extent to which the intervention was delivered as planned by the instructor** | **The type of adverse events** | **The number of adverse events** |
| --- | --- | --- | --- | --- | --- | --- |
| **CERT item** | **5, 16a** | **16b** | **16a** | **16b** | **11** | **11** |
| **CONSORT E-Health item** | **-** |  | **-** | **-** | **-** | **-** |
| **Labat, 2022, France [52]** | Not described | The control group lost to follow up was n=14 at 12 weeks, n=13 at 24 weeks, and n=2 at 36 weeks. Only 24 participant completed the study in NTG group  The intervention group lost to follow up was N=8 at 12 weeks, n=10 at 24 weeks, and n=5 at 36 weeks. Only 32 participants completed the study in the TG group. | Not described | Not described | Not described | Not described |
| **Plumb Vilardage, 2022, United States [44]** | Session attendance. | All participants assigned to the Engage-PA intervention condition completed both sessions (100% treatment completion). | Not described | Not described | Not described | Not described |
| **Ostlind, 2021, Sweden [43]** | For each participant, adherence to Fitbit-use during the entire period was calculated and presented as the percentageof valid days (> 1,500 steps) during the study period. The adherence to Fitbit-use per week was alsocalculated by dividing the number of valid days per week by seven (days in a week). For the whole group, adherenceto Fitbit-use was calculated as mean of the percentage of adherence for the entire period and for each week. | Sixty-seven participants completed the maximum numberof days (84) whereas eight participants finished the intervention prematurely due to practical and technical reasons. They participated during a total number of days of 63–83 days with a mean value of 73 days. The Fitbit was used on average 88.4 % (SD 11.6) of the days during the 12-week intervention with highest adherencein week 2 (94.7 %) and lowest in week 12 (80.5 %). The adherence to Fitbit-use decreased gradually over the 12weeks (β-coefficient − 1.3, 95 % CI -1.8 to -0.8, p = <0.001). | Not described | Not described | Not described | Not described |
| **Christiansen, 2020, United States [48]** | The participant’s ability to adhere to the intervention (monitoring steps/day). We classified adherence as “achieved” for participants who had ≥80% of the weekly steps/day goal recorded by the physical therapist and “not achieved” for those with <80% of the weekly steps/day goals recorded. | On average, participants in the intervention group attended a mean ± SD 20 ± 8 PT sessions for a mean ± SD 10.4 ± 5.5 weeks, and participants in the control group attended a mean ± SD 18 ± 7 PT sessions for mean ± SD 9.0 ± 2.7 weeks. Sixty percent (12 of 20 participants) of those in the intervention group monitored steps/day at least 80% of the time while in PT. | Including the physical therapist’s adherence to administering the intervention (establishing a weekly steps/day goal with the participant). Adherence to administering the weekly steps/day goal by the physical therapist was measured by a research assistant counting the number of goals documented in the home exercise program log from baseline to discharge from PT. | 45% of physical therapists (9 of 20) were in adherence with the administration of the intervention by documenting weekly steps/day goals ≥80% of the time. | Not occurred | Not occurred |
| **Li, 2020a, Canada [38]** | (1) Attended the education session,  (2) Used their Fitbit ≥5 days per week in ≥11 weeks (3) Participated in ≥3 of 4 counseling calls. | Intervention adherence in the intervention group was 100% (26/26) for education session attendance, 96% (25/26) for PT counseling phone calls, and 81% (21/26) for Fitbit use. In all, 81% (21/26) of participants met all 3 fidelity criteria. Adherence rates were similar in the delayed group when participants received the program in week 13. | Not described | Not described | a) Muscle pain  b) Falls | a) 7 (5 intervention group, 2 delayed group) b) 6 (3 intervention group, 3 delayed group) |
| **Li, 2020b, Canada [53]** | a) Attended the education session b) Used their Fitbit ≥5 days/week in at least 7 weeks (Fitbit use was defined as participants having steps recorded in their Fitbit within a 24-hour duration) c) Participation in at least 3 of 4 counseling calls. | The average intervention adherence in the immediate group was 98.3% for the education session attendance, 88.1% for the physical therapist phone calls, and 83.1% for Fitbit use (Table 5). In all, 78% of participants met all 3 fidelity criteria. | Not described | Not described | a) Muscle pain  b) Ligament sprain | a) 19 ( 10 intervention group, 9 delayed group) b) 4 (3 intervention group, 1 delayed group) |
| **Zaslavsky, 2019, United States [47]** | Fitbit wear time, which was used as a proxy for adherence to wearing and syncing the Fitbit device with the mobile app. | One participant could not finish the intervention due to development of a major depressive episode, and one participant did not contribute week 19 sleep data due to a scheduled surgical procedure, which left a total of 22 participants who completed the full protocol and a 96% retention rate. | An interventionist, who delivered phone calls,  received training in Motivational Interviewing.  All phone calls were scripted, recorded and randomly sampled as to ensure the intervention fidelity. | Not described | Not described | Not described |
| **Li, 2018, Canada [40]** | Not described | Not described | Not described | Not described | No adverse event associated with the intervention (e.g., falls, cardiovascular and musculoskeletal events) was reported by participants during the study. | No adverse event associated with the intervention (e.g., falls, cardiovascular and musculoskeletal events) was reported by participants during the study. |
| **Paxton, 2018, United States [50]** | Intervention adherence (percentage days of successful real-time physical activity data collection in physical activity feedback; mean ± SD), and dose goal attainment (percentage of participants meeting weekly physical activity dose goals). | Adherence to daily use of the Fitbit sensor and peripheral monitors was 92.3% as tracked by remote assessment of use by the study coordinator. Dose goal attainment of the individualized weekly goals was attained 65% of the time overall, with 65% of participants achieving their goals in the first week of intervention and 57% achieving weekly goals in Week 12. | Not described | Not described | Not described | Not described |
| **Darabseh, 2017, Jordan [49]** | Not described | Not described | Not described | Not described | Not described | Not described |
| **Katz, 2017, United States [51]** | Adherence for the intervention groups was estimated by the proportion of study days for which steps were recorded in the diaries. | Overall, steps were recorded on 88.8% of study days (92.6% for intervention group 1 and 85.1% for intervention group 2). | Not described | Not described | Calf muscle strain | 1 |
| **Li, 2017, Canada [39]** | Adherence to the study protocol. | All but 1 participant adhered to the intervention protocol. | Not described | Not described | Not occurred | Not occurred |
| **Skrepnik, 2017, United States [45]** | A post study adherence check in intervention group 1 patients occurred at day 180, when data were downloaded from the app (no visit). | Of the patients in intervention group 1 who entered the 90 to 180 days adherence period, 36/101(35.6%) were 80% or more compliant with use of the OA GO app. | Not described | Not described | No major adverse events or treatment-emergent serious adverse events related to thedevice or protocol occurred. Treatment-emergent adverse events:arthralgia and upper respiratory tract infection. | No major adverse events or treatment-emergent serious adverse events related to thedevice or protocol occurred. Two treatment-emergent adverse events. |
| **Hiyama, 2011, Japan [37]** | Not described | No participant dropped out during the intervention period due to exacerbation of pain. | Not described | Not described | Not described | Not described |
| **Ng, 2010, Australia [41]** | Not described | Of the participants who enrolled, 77% completed the study (three-day group: n = 13, five-day group: n = 15). Three participants dropped out during the first six weeks of the study, before the walking program began. Reasons were a death in the family (n = 1), a physician’s advice to withdraw due to potential impact of walking on OA (n = 1) and a physician’s advice to withdraw due to potential impact of walking on other health conditions (n = 1). Five additional participants dropped out during the walking program. Reasons for drop-out from the three-day walking group were a death in the family (n = 1; dropout in Week 8), pain in the knees (n = 1; Week 7) and a torn Achilles tendon (n = 1; Week 7), and from the five-day walking groups were pain while walking due to leg length discrepancies (n = 1; Week 12) and development of Bakers’ Cyst causing pain while walking (n = 1; Week 9). | Not described | Not described | Not described | Not described |
| **Talbot, 2003, United States [46]** | Educational adherence was defined as the number of arthritis self-management program classes attended divided by the total number of classes. For the home-based pedometer group, adherence was defined as the number of steps recorded on the daily pedometer log that met or surpassed the weekly step goal. | Average adherence to wearing the pedometer and recording on the log daily was 76%. Compliance rate for meeting the step goal was 48%, ranging from a high of 56% (Weeks 4–8) to a low of 35% (Weeks 9–12). Class attendance for the arthritis self-management course was 71% for the entire group (Walk group : 66%; Education group: 77.1%, P=0.18). | Not described | Not described | Not described | Not described |

*Abbreviations and explanatory:
PA=Physical Activity, PT=Physical Therapy, OA= Osteoarthritis, SD=Standard Deviation, n=number, CI=Confidence Interval.*
