# Supplementary material for: Reporting quality of interventions using a wearable activity tracker to improve physical activity in patients with inflammatory arthritis or osteoarthritis: a systematic review
Source: Rheumatol Int. 2022 Dec 1;43(5):803–24. doi: 10.1007/s00296-022-05241-x (PMC10073167; doi:10.1007/s00296-022-05241-x)
Supplement: Supplementary file 6 — Supplementary file6 (DOCX 123 KB) [file 296_2022_5241_MOESM6_ESM.docx]

Article title: Reporting quality of interventions using a wearable activity tracker to improve physical activity in patients with inflammatory arthritis or osteoarthritis: a systematic review

Journal: Rheumatology International

M.A.T. van Wissen^1^*, M.A.M. Berger^2^, J.W. Schoones^3^, M.G.J. Gademan^1, 4^, C.H.M. van den Ende^5,6^, T.P.M. Vliet Vlieland^1^, S.F.E. van Weely^1^

1.Department of Orthopaedics, Rehabilitation and Physical Therapy, Leiden University Medical Center, Leiden, The Netherlands; 2.The Hague University of applied sciences, The Hague, The Netherlands; 3. Directorate of Research Policy (Walaeus Library), Leiden, The Netherlands;4. Department of Clinical Epidemiology, Leiden University Medical Center, Leiden, The Netherlands; 5. Department of Research, Sint Maartenskliniek, Nijmegen, The Netherlands; 6.Department of Rheumatology, Radboud University Medical Center, Nijmegen, The Netherlands

*Corresponding author: M.A.T. van Wissen. m.a.t.van_wissen@lumc.nl

**Supplementary Table 6 Risk of Bias Assessment Rob-2 tool: randomized controlled trials in a systematic review on interventions promoting PA in patients with inflammatory arthritis or osteoarthritis**

|  | D1 | D2 | D3 | D4 | D5 | Overall |
| --- | --- | --- | --- | --- | --- | --- |
| Labat, 2022, France [52] |  |  |  |  |  |  |
| Christiansen, 2020, United States [48] | \|  \| \| --- \| |  |  |  |  |  |
| Ostlind, 2021, Sweden [43] |  |  |  |  |  |  |
| Li, 2020a, Canada [38] |  |  |  |  |  |  |
| Li, 2020b, Canada [53] |  |  |  |  |  |  |
| Li, 2018, Canada [40] |  |  |  |  |  |  |
| Paxton, 2018, United States [50] |  |  |  |  |  |  |
| Katz, 2017, United States [51] |  |  |  |  |  |  |
| Darabseh, 2017, Jordan [49] |  |  |  |  |  |  |
| Li, 2017, Canada [39] |  |  |  |  |  |  |
| Skrepnik, 2017, United States [45] |  |  |  |  |  |  |
| Hiyama, 2011, Japan [37] |  |  |  |  |  |  |
| Talbot, 2003, United States [46] |  |  |  |  |  |  |

|  |  |
| --- | --- |
| \|  \| \| --- \| | Low risk |
| \|  \| \| --- \| | Some concerns |
| \|  \| \| --- \| | High risk |
|  |  |
|  |  |

*Abbreviations and explanatory: D1 = Randomisation process; D2 = Deviations from the intended interventions; D3 = Missing outcome data; D4 = Measurement of the outcome; D5 = Selection of the reported result.*
