# Supplementary material for: Reporting quality of interventions using a wearable activity tracker to improve physical activity in patients with inflammatory arthritis or osteoarthritis: a systematic review
Source: Rheumatol Int. 2022 Dec 1;43(5):803–24. doi: 10.1007/s00296-022-05241-x (PMC10073167; doi:10.1007/s00296-022-05241-x)
Supplement: Supplementary file 7 — Supplementary file7 (DOCX 49 KB) [file 296_2022_5241_MOESM7_ESM.docx]

Article title: Reporting quality of interventions using a wearable activity tracker to improve physical activity in patients with inflammatory arthritis or osteoarthritis: a systematic review

Journal: Rheumatology International

M.A.T. van Wissen^1^*, M.A.M. Berger^2^, J.W. Schoones^3^, M.G.J. Gademan^1, 4^, C.H.M. van den Ende^5,6^, T.P.M. Vliet Vlieland^1^, S.F.E. van Weely^1^

1.Department of Orthopaedics, Rehabilitation and Physical Therapy, Leiden University Medical Center, Leiden, The Netherlands; 2.The Hague University of applied sciences, The Hague, The Netherlands; 3. Directorate of Research Policy (Walaeus Library), Leiden, The Netherlands;4. Department of Clinical Epidemiology, Leiden University Medical Center, Leiden, The Netherlands; 5. Department of Research, Sint Maartenskliniek, Nijmegen, The Netherlands; 6.Department of Rheumatology, Radboud University Medical Center, Nijmegen, The Netherlands

*Corresponding author: M.A.T. van Wissen. m.a.t.van_wissen@lumc.nl

**Supplementary Table 7 Risk of Bias assessment ROBINS-I tool: non-randomized controlled trials in a systematic review on interventions promoting PA in patients with inflammatory arthritis or osteoarthritis**

|  | Low risk |
| --- | --- |
|  | Some concerns |
|  | High risk |

|  | D1 | D2 | D3 | D4 | D5 | D6 | D7 | Overall |
| --- | --- | --- | --- | --- | --- | --- | --- | --- |
| Plumb Vilardage, 2022, United States [44] |  |  |  |  |  |  |  |  |
| Zaslavsky, 2019, United States [47] |  |  |  |  |  |  |  |  |
| Ng, 2010, Australia [41] |  |  |  |  |  |  |  |  |

*Abbreviations and explanatory: D1 = Focusing on confounding; D2 = Selection of participants into the study; D3 = Classification on interventions; D4 = Deviations from intended interventions; D5 = Missing data; D6 = Measurements of outcomes; D7 = Selection of the reported results.*
